# SOMmelier—Intuitive Visualization of the Topology of Grapevine Genome Landscapes Using Artificial Neural Networks

**DOI:** 10.3390/genes11070817

**Published:** 2020-07-17

**Authors:** Maria Nikoghosyan, Maria Schmidt, Kristina Margaryan, Henry Loeffler-Wirth, Arsen Arakelyan, Hans Binder

**Affiliations:** 1 Research Group of Bioinformatics, Institute of Molecular Biology of National Academy of Sciences RA, Yerevan 0014, Armenia; m_nikoghosyan@mb.sci.am (M.N.); aarakelyan@sci.am (A.A.); 2 Institute of Biomedicine and Pharmacy, Russian-Armenian University, Yerevan 0051, Armenia; 3 Interdisciplinary Centre for Bioinformatics, University of Leipzig, 04107 Leipzig, Germany; schmidt@izbi.uni-leipzig.de (M.S.); wirth@izbi.uni-leipzig.de (H.L.-W.); 4 Research Group of Plant Genetics and Immunology, Institute of Molecular Biology of National Academy of Sciences RA, Yerevan 0014, Armenia; kristinamargaryan@ysu.am; 5 Department of Genetics and Cytology, Yerevan State University, Yerevan 0025, Armenia

**Keywords:** grapevine genomes, genetic diversity, dissemination of vine, genome passporting, self-organizing maps, genome portrayal

## Abstract

Background: Whole-genome studies of vine cultivars have brought novel knowledge about the diversity, geographical relatedness, historical origin and dissemination, phenotype associations and genetic markers. Method: We applied SOM (self-organizing maps) portrayal, a neural network-based machine learning method, to re-analyze the genome-wide Single Nucleotide Polymorphism (SNP) data of nearly eight hundred grapevine cultivars. The method generates genome-specific data landscapes. Their topology reflects the geographical distribution of cultivars, indicates paths of cultivar dissemination in history and genome-phenotype associations about grape utilization. Results: The landscape of vine genomes resembles the geographic map of the Mediterranean world, reflecting two major dissemination paths from South Caucasus along a northern route via Balkan towards Western Europe and along a southern route via Palestine and Maghreb towards Iberian Peninsula. The Mediterranean and Black Sea, as well as the Pyrenees, constitute barriers for genetic exchange. On the coarsest level of stratification, cultivars divide into three major groups: Western Europe and Italian grapes, Iberian grapes and vine cultivars from Near East and Maghreb regions. Genetic landmarks were associated with agronomic traits, referring to their utilization as table and wine grapes. Pseudotime analysis describes the dissemination of grapevines in an East to West direction in different waves of cultivation. Conclusion: In analogy to the tasks of the wine waiter in gastronomy, the sommelier, our ‘SOMmelier’-approach supports understanding the diversity of grapevine genomes in the context of their geographic and historical background, using SOM portrayal. It offers an option to supplement vine cultivar passports by genome fingerprint portraits.

## 1. Introduction

Grapes are not only tasty, but also one of the most economically and culturally important crops. They are used for both winemaking and fresh (‘table’) consumption. Grape is unique, not only because it is a major global perennial crop, but also because of its historical and cultural connections with the development of humans. According to the OIV (International Organization of Vine and Wine) the surface area of the world vineyard is estimated at 7.4 million hectares, with a production of 76 million tons of fresh grapes and 260 million hectoliters of wine (http://www.oiv.int/). Grapevine (*Vitis Vinifera*) is one of the oldest of the cultivated plants for which living progenitors still exist. The broad geographic area of the wild grapes and a bewildering number of different forms expanding from Europe to Asia and Caucasus continue to intrigued researchers, with a crucial question about the origin and domestication of the grape. Archaeological and historical studies suggested that the cultivation of the domesticated grape (*V. vinifera* L. subsp. sativa) started about 10,000 to 8000 years ago, from its putative wild ancestor (*V. vinifera* L. subsp. sylvestris), and the primary center of domestication was located between the Near East [1] and the Transcaucasian region [2]. These regions were populated by the Neolithic Shulaveri-Shomu, and later Kura-Araxes cultures, in the South Caucasus and Fertile Crescent regions, ranging from (today’s) Georgia, through Azerbaijan, Armenia, northern Iran and eastern Anatolia [3]. Later, grapevine disseminated into the Southern Balkans and East Mediterranean Basin, then to the Western Europe and, finally, domesticated grapes were introduced to Central Europe during the first millennium BCE [4]. Hereby, wine grapes have followed human civilization from South Caucasus, southwards and westwards, spread first by seafaring Phoenicians and Greeks, and later affected by Roman and Ottoman Empires throughout the Mediterranean world [5]. During its spreading across the regions, grapes slowly mutated and adapted to their environments. The slow divergence over thousands of years in combination with the spontaneous hybridization, somatic variation and selection by humans created the incredible diversity of the more than 6000 cultivated varieties, which, in contrast to its wild progenitor, is more diverse and heterozygous according to OIV (http://www.oiv.int/). Different authors evidenced the presence of secondary domestication centers, where spontaneous hybridizations among wild plants and cultivated forms or targeted selection, created the pattern of the modern Western European cultivars [4]. M.A. Negrul, an outstanding pioneer researcher of vine, subdivided the varieties based on geographic origin and morpho-ecological traits into the ecotypes occidentalis (France, Germany, Spain, Portugal), pontica (Asia Minor, Romania, Hungary, Greece, Georgia, Bessarabia) and orientalis (Armenia, Iran, Afghanistan, Azerbaijan and Central Asia), where the primary center of a given plant is the region of the richest genetic diversity [6].

In the last years, whole-genome studies of vine cultivars using genotyping and sequencing technologies have added novel knowledge about the diversity, geographical relatedness, historical origin, phenotype associations, genetic markers and distribution paths of the vine [4,7,8,9,10,11]. Genetic relatedness between cultivated (*V. Vinifera* L. subsp. sativa) and wild (*V. Vinifera* L. subsp. sylvestris) grapes suggest at minimum two separate domestication events, one derived from the Transcaucasian wild grape and another one in Western Europe, where cultivars experienced introgression from local wild grape [4,7]. Associations between candidate genes and important agronomic traits, such as berry shape and aromatic compounds, provide possible genetic targets for grapevine improvement [9]. Breeding for larger berries has been related to genetic signatures of positive selection for table grapes in geographic regions, where alcohol was prohibited by religious rules [8]. The further impact of genomic studies will depend on the understanding of the genotype-phenotype associations, especially for complex traits governed by polygenic architecture, genotype-environment interactions in different geographic regions [11]. These tasks challenge activities and research infrastructure for collecting cultivar accessions, sequencing and data deposition, for providing phenotype-genotype databases and vine passporting projects, and, last but not least, the development of bioinformatics methods and tools which enable an analysis of these data, in terms of knowledge mining and feature extraction, in an easy and intuitive fashion.

Here, we apply SOM (self-organizing maps) portrayal [12], a neural network-based machine learning method with strong visualization capabilities to genome-wide Single nucleotide polymorphism (SNP) data of nearly eight hundred grapevine cultivars collected from Middle Asia in the East to Iberian peninsula in the West and from overseas regions [11]. SOM portrayal has been developed by us for the detailed analysis of high-dimensional omics data, including diversity and developmental issues, feature selection, knowledge mining and phenotype association of transcriptomic [13,14,15], epigenetic [16,17], proteomics [18] and genetic data [19], and of combinations of them [20]. In the context of plants, SOM-portrayal has been previously applied by us for the typing of algae of the genus Prototheca [21] and by others for studying early seed maturation in garden pea [22]. This work aims at providing a prototypic application of SOM machine learning and linked downstream analytics to illustrate its potency for studying plant genomes.

Strengths of the applied method are dimension reduction and visualization capabilities [23]. Particularly, our method generates accession-specific images of the data landscapes. These personalized omics-portraits provide options for the intuitive evaluation of feature space, and for mutual comparisons between the individual accessions. The recent application of SOM-portrayal to population-level distributions of disease-related human SNP-variants demonstrated its capability to extract genetic features and to describe genetic diversity, based on the topology of the SNP-landscapes [19].

This study aims at applying SOM-portrayal to the genetics of vine, in order to generate SNP-landscape of grape genotypes, to relate its topology to the geographical distribution, to possible paths of cultivar distribution, to selected genetic markers, and link them to grape utilization. In gastronomy, the sommelier is the wine ‘waiter’ (or ‘waitress’), who recommends combining wine with food based on traditional knowledge about the character of wine varieties, their taste and geographic origin, and thus to support the decision-making of guests for a successful dinner. In analogy, our ‘SOMmelier’-approach aims at supporting understanding genetic diversity and relatedness of grapevine genomes in the context of their geographic and historical background by application of SOM portrayal.

## 2. Materials and Methods

### 2.1. Data and Preprocessing

*Grape* cultivars data: grapevine genetic SNP- and phenotype data were taken from [11]. The data set consists of 783 grapevine samples originated from 4 grapevine collections (Table S1, Passport data of 783 cultivars included in the study) collected from eleven geographic regions, ranging from Middle Asia to Iberia and New World accessions. The genotype data matrix of 10,207 SNPs for 783 unique samples was taken from https://urgi.versailles.inra.fr/Species/Vitis/Data-Sequences/Genotyping-data repository.

Human population data: For a comparison of genomic relatedness between worldwide geographic strata, we analyzed genome-wide microarray SNP data (Illumina 650Y arrays) from the Human Genome Diversity Project (HGDP) [24], as described previously [19]. This data consists of 650,0000 SNPs for 940 individuals from 8 geographical regions (Africa, Europe, Middle East, South and Central Asia, East Asia, Oceania, and America). Genotypes for 99 individuals of Armenian ethnicity (Illumina Human Omniexpress microarray platform) were taken from a publication by Haber et al. [25]

### 2.2. Conceptual Overview

Our analysis concept is based on the following main ingredients: (i) transformation of SNP data into excess minor allele frequencies (eMAF), which increases sensitivity for subtle differences between the genomes of the vine accessions; (ii) dimension reduction of the more than 10,000 SNP data into a matrix of 2500 so-called ‘meta-SNPs’, using unsupervised clustering and their visualization in terms of individual accession portraits using SOM machine learning. (iii) further reduction of dimension by segmentation of the SNP-portraits into about one-to-two dozen so-called spot clusters of correlated SNPs, which serve as fingerprint features of the vine genomes; (iv) diversity analysis of accessions using different approaches (principal component analysis, correlation networks, t-SNE, minimum spanning tree). Each of the approaches used enables discovering different aspects of the mutual relatedness between the cultivars. (v) So-called pseudotime analysis as a special type of diversity analysis to analyze genetic similarities in terms of multibranched trees with possible impact for temporal diversification of vine cultivars; (vi) phenotype associations to map information of vine utilization on the SNP landscapes.

### 2.3. Allele Coding and SNP-Score

In order to further process the data, we coded the genotype of each SNP using a trinary code, with ‘0′ for homozygous major alleles (AA), ‘1′ for heterozygous alleles (Aa and aA), and ‘2′ for homozygous minor alleles (aa).

First, let us consider one SNP in the data set of N cases, e.g., of N =783 cultivar samples. The fractions of the three genotypes of this SNP are defined as p_ij_ = N_ij_/N (i,j = A, a), where N_ij_ is the number of SNPs with the respective allele in the data set. The minor allele frequency of a SNP is MAF = 2 p_aa_ + 1 (p_aA_ + p_Aa_) with p_aa_ < p_AA_. With the SNP-code introduced above one finds for its mean value averaged over all cases, <SNP-code> = p_AA_ * 0 + (p_Aa_ + p_aA_) * 1 + p_aa_ * 2 = 2 MAF, i.e., the mean SNP-code of a SNP equals twice its MAF. For further data processing, we define a SNP-score by centralizing the SNP-code of each SNP with respect to its mean value, SNP-score = SNP-code – 2 MAF. One obtains SNP-score = −2 MAF for the major allelic SNPs, SNP-score = 1 – 2 MAF for heterozygous SNPs and SNP-score = 2 (1 – MAF) for the minor allelic SNPs.

Second, let us consider a group of n correlated SNPs in the data set, and calculate the mean of their SNP-score in one selected sample. In the next subsection, we show that such a group of SNPs is given, e.g., by a meta-SNP, or a ‘spot-module’ as obtained below in SOM-analysis. Analogous consideration as above deliver the result for the group-averaged SNP-score, <SNP-score> = 2 (MAF - <MAF>) where <…> here denotes group averaging referring to one selected sample. Hence, the group-averaged SNP-score estimates the deviation of the mean MAF of the considered SNPs, in a certain sample from their mean in the considered population. Accordingly, it can be understood as an excess MAF-value (eMAF), where positive SNP-scores mean higher frequencies than in the population while negative values refer to reduced frequencies. The centralization of each feature (here the SNP-code of each SNP) with respect to its mean value averaged over all samples is applied as a standard preprocessing step in SOM analysis. Features space of centralized values is more sensitive to subtle differences between samples than the feature space of non-centralized values.

### 2.4. SOM Portrayal and Spot Detection

SOM-portrayal of SNP data was performed as described previously [19]. In short: our SOM implementation used a ternary code with the values 0, 1 and 2 for major homozygous, heterozygous and minor heterozygous genotypes, respectively, as introduced above. Next, SNP data were mean centralized and then clustered using the self-organizing map (SOM) machine learning. SOM training translates the original data matrix consisting of the allele scores of N = 10,206 SNPs collected from M = 783 cultivar accessions into a data matrix of reduced dimensionality of K = 2500, so-called meta-SNP profiles. Hereby, the term ‘profile’ denotes the vector of eMAF score values across the cultivars. The SOM training algorithm distributes the SNPs over the K micro-clusters of meta-SNPs, by minimizing the *Euclidean* distance between the SNP- profiles as a similarity measure. It ensures that SNPs with similar profiles cluster together in the same or in closely located meta-SNPs. Each meta-SNP profile can be interpreted as the mean profile averaged over all SNP profiles of the respective meta-SNP cluster. For each cultivar accession, the meta-SNP score obtained provides the excess MAF (eMAF, see above). It is defined as the difference between the mean minor allele frequency (MAF of meta-SNP) of all SNPs collected in this meta-SNP in this particular sample, minus the mean MAF of these SNPs averaged over all samples. The eMAF values of each cultivar accession are visualized by arranging them into a two-dimensional M = 50 × 50 grid, and by using a red to blue color-code for maximum to minimum eMAF-values in each of the grid images. These images ‘portray’ the genetic landscape of each accession studied in units of the eMAF SNP-score. We used SOM as implemented in the “oposSOM” R package [23]. Cultivars were labelled according to the geographical region where they were selected (see [4] for details) and, alternatively, using different cluster assignments (see below). Mean SNP-SOM portraits of cultivars from the same geographic regions were obtained by averaging the meta-SNP values of the respective individual SNP-portraits. The self-organizing properties of the SOM algorithm generates red spot-like regions referring to correlated SNP-profiles showing high eMAF-values in the respective accession portraits. We used segmentation algorithms developed previously [23] to extract so-called spot-clusters from these (red) regions. Each of these spot-clusters includes hundreds of SNPs.

### 2.5. Phenotype Association, Accession Diversity, Pseudotime Analysis and Clustering

Categorical phenotypes such as the utilization of grapes for table or wine usage were associated with the SOM SNP-landscape by performing ANOVA (analysis of variance) of SNP-metagenes of the sub-collection of cultivars of a certain phenotype (e.g., of all cultivars of table-vine usage) and coloring the meta-SNP pixels in the SOM according to the obtained p-value (in units of -log(p)), from red (high) to blue (low). Phenotype correlation maps were obtained by calculating the point biserial correlation between the eMAF profile of each metagene and the respective phenotype profile, which is given, e.g., for ‘table vine utilization’ by categorical values ‘0′ (no table usage) and ‘1′ (table usage). The metagenes of the map were then colored between red (maximum correlation) and blue (minimum correlation). Point serial correlation de facto provides difference portraits between cultivars of the respective phenotype and all others, e.g., between the mean portrait of cultivars of wine utilization, minus the mean portraits of cultivars of non-wine utilization.

Accession diversity analysis was performed based on meta-SNPs using principal component analysis (PCA), similarity net and minimum spanning tree plots, based on correlation metrics between the individual SOM-portraits as implemented in oposSOM [23]. t-SNE (t-distributed Stochastic Neighbour Embedding), URD-plots and pseudotime plots were generated using the program ‘URD’ [26]. URD estimates multibranched developmental trajectories based on mutual similarities between the accessions, a k-nearest neighbor graph presentation and directed (from ‘root’ to ‘tip’) simulation of a diffusion-like process. It provides a pseudotime (PT) value between zero and unity for each accession, where values near zero mean closer similarity to the root accessions, and values near unity mean closer similarity to tip-accessions. URD generates branched tree structure by joining diffusion trajectories passing through the same accessions.

Grapevine cultivars were stratified according to the geographic region in agreement with [10], where they were collected and using an eight cluster C1–C8 (C-clusters) division, according to [11]. Independent ‘t-SNE’ clustering was performed using the Jaccard-algorithm implemented in ‘URD’ software [26], which provided ten clusters (see below).

## 3. Results

### 3.1. SOM Portrayal of Cultivars among Geographical Regions

**Figure 1:**
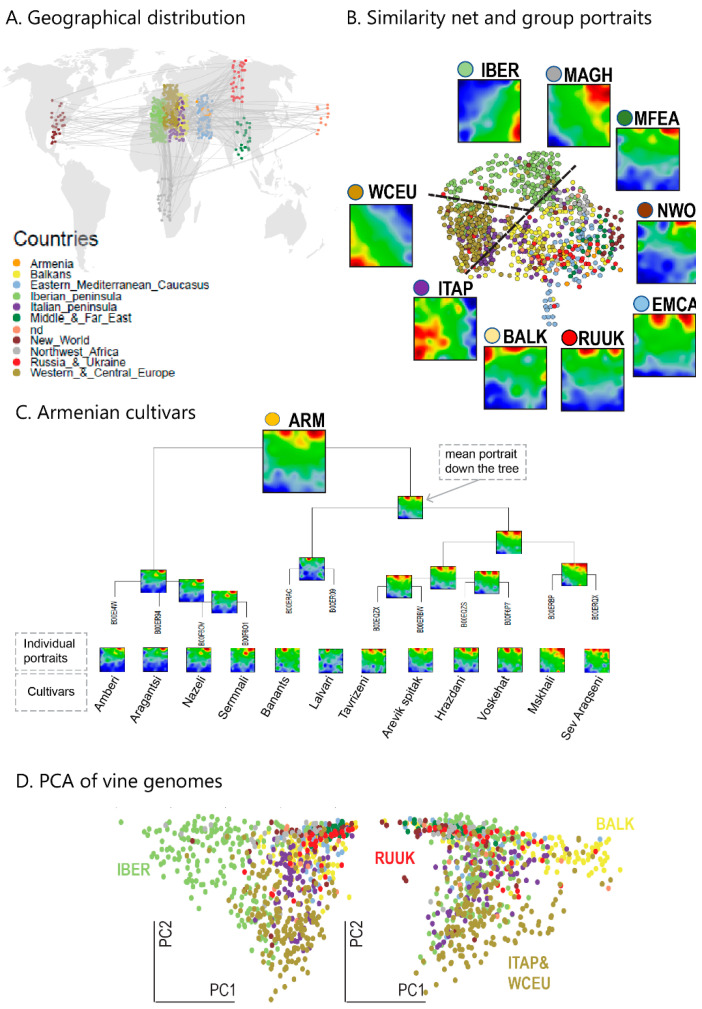
Self-organizing maps (SOM) portrayal of grapevine Single Nucleotide Polymorphism (SNP)-data: (**A**) Sampling of grapevine cultivars from different geographic regions. The thin lines visualize minimum spanning relations between the samples. (**B**) SOM-portraits of cultivars from different geographic regions indicate specific spot patterns of SNPs. The similarity net shows three major clusters of cultivars originating from the Iberian Peninsula (IBER), western and central Europe (WCEU) and Italian Peninsula (ITAP), and the other accessions originating mostly from Eastern Europe, Maghreb, Near East and Middle Asia (see dashed lines). (**C**) Hierarchical clustering tree of the cultivars originated from Armenia. The SOM portraits were progressively averaged in upwards direction. The mean portrait shows the weighted average of the spot patterns of the individual portraits. (**D**) Principal component plots reveal the distribution of accessions from WCEU (and from the Italian peninsula, ITAP) and IBER, along the first two principal components PC1 and PC2, respectively, and the split of the samples from Russia and Ukraine (RUUK) and the Balkan (BALK) along PC3. Color code of samples is provided in part B.

SOM-training of the SNP-data of 783 grapevine cultivars provided a SNP-portrait of each accession. Each portrait represents a two-dimensional pixel-plot, where each of the pixels, the so-called meta-SNP, is colored according to its excess minor allele frequency (eMAF) of correlated meta-SNPs in the chosen cultivar (Additional File 1). According to their origin, the SNP-portraits were stratified into nine geographic regions, ranging from Middle and Far East (MFEA), Eastern Mediterranean and Caucasus (EMCA) over Russia and Ukraine (RUUK), Balkan (BALK), Western and Central Europe (WCEU), Italian Peninsula (ITAP) to Maghreb (MAGH) and Iberia (IBER) in the ‘Old World’ (Figure 1A). Cultivars from Armenia (ARM) and from the New World (NEWO), including America, Australia, New Zealand and South Africa, were separately considered (see Abbreviations for glossary of geographic regions). The mean SNP-portrait of each region reveals specific patterns of red spot-like areas which visualizes the respective genotype in terms of the ‘meta-SNP’ score landscape, where red/blue areas refer to positive/negative eMAF values, meaning that the mean minor allele frequency (MAF) of the SNPs in this spot from this region exceeds/falls below their mean MAF value averaged over all cultivars studied of all geographic regions (Figure 1B). The diversity of cultivars from Armenia and the portrait averaging applied is illustrated in terms of a hierarchical clustering tree (Figure 1C). The vine accessions roughly split into three major groups in the similarity net (Figure 1B), namely into cultivars from WCEU (and ITAP), from IBER and from the other regions, where the former two groups distribute along the first two principal component PC1 and PC2 of principal component analysis, while PC3 segregates cultivars of BALK from RUUK, MFEA and EUCA (Figure 1D).

### 3.2. The Topology of SNP-Landscapes Characterizes the Diversity of Cultivars

**Figure 2:**
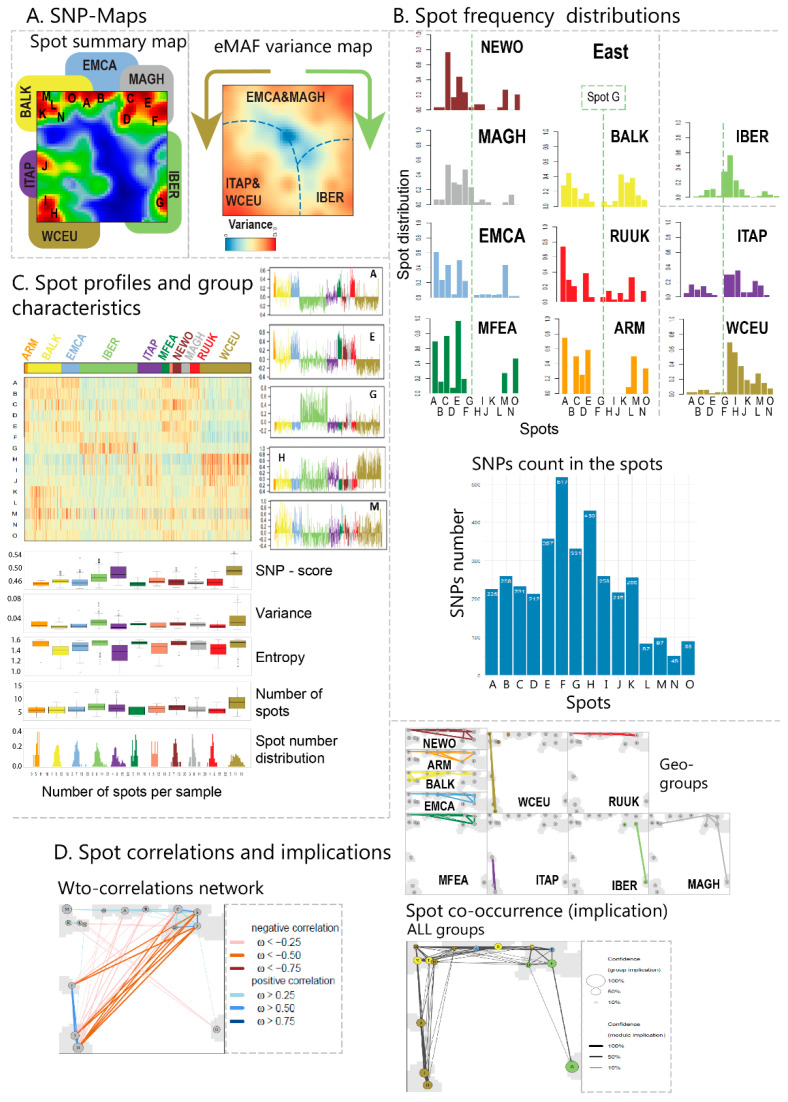
SNP-spot characteristics: (**A**) The SNP-maps provide information about SNP-spots (spot summary map) and variance of the allele-score (variance map). The maps roughly divide into three main areas, accumulating cultivars from WCEU, IBER and the ‘East’ (i.e., BALK, RUUK, MFEA, EMCA, MAGH). (**B**) Spot frequency distributions of different geographic regions assign region-specific spots, namely around G (vertical dashed line) for IBER, to the right from G (spots H–K) for WCEU and ITAP and to the left (spots A–E) for vines from the East. BALK and RUUK vines show mixed properties of WCEU and East vines. The number of SNPs per spot is largest in spots E–H. (**C**) SNP-score profiles of the spots and their geographic group-wise characteristics (mean eMAF, variance, entropy, mean spot number and spot number distributions). (**D**) Correlations between spot profiles and co-occurrence (implication) of spots. Correlations were calculated using the weighted topology-overlap (WTO) algorithm [27]. Implications were shown for all groups and separately for each of the geographic groups. The lines link spots which frequently co-occur and thus imply each other due to associations between the SNPs covered by them [13].

The inspection of the individual accession portraits (Additional File 1) reveals a high diversity of textures. They reflect the allelic landscapes in terms of areas of positive and negative eMAF values, colored in red and blue, respectively, referring to enriched or depleted minor allelic genotypes. The spot summary map collects the ‘red’ spots most frequently observed in the individual maps (Figure 2A). It provides the SNP-landscape of the accessions studied by distributing the SNPs across the x-y-plane, making use of the self-organizing properties of SOM machine learning, and then, plotting the respective eMAF values along the ‘height’ dimension. The topology of this landscape is given by the eMAF-height profile. Overall, we identified fifteen such spots labelled with capital letters A–O. Overall, they agree with the spot patterns observed in the mean portraits of the geographic regions considered (Figure 2B). Lists of SNPs collected in the spots are provided in Additional File 2. The ‘variance map’ indicates that the spot regions contain SNPs of the largest variance of their eMAF SNP-score among the cultivars. The maps can be divided into three major regions of spots observed predominantly in the SNP-portraits of IBER, WCEU and ‘the East’ (from Central Europe), respectively (Figure 2A). The spot frequency distributions of the different geographical regions clearly reflect these three different patterns, in terms of bar plots centered around spot G frequently expressed in grapevine cultivars from IBER (see the vertical dashed line for orientation). In contrast, cultivars from WCEU and ITAP frequently express spots H–J (to the right from G), while grapevine cultivars from the ‘East’ frequently express spots A–F located to the left from spot G. BALK and RUUK cultivars reveal intermediate patterns. Most SNPs, namely between 300 and 550 per spot, were included in spots E–H, while each of the remaining ones contains about 200 SNPs.

The spot-profiles show the mean values of the eMAF-scores averaged over all meta-SNPs included in the respective spot among the cultivars and, particularly, their region-specific alterations (Figure 2C). Largest SNP-score values, and thus high excess frequencies of the minor alleles included in the spot, were observed for WCEU and ITAP, followed by IBER and the ‘East’ (see boxplots below the heatmap in Figure 2C). Hence, one finds a tri-partition of accessions with respect to the amplitude of the SNP-score as found also for spot-distributions. The variance of the SNP-score (and entropy, which estimates the degree of randomness of spot patterns) is largest for WCEU and IBER, and to a less degree MFEA and EMCA, paralleled by the largest number of spots per accession, reflecting the largest genetic diversity in these regions (see also the spot-number distribution in the last row of plots in Figure 2C), which are assumed to serve as criteria of centers of vine cultivation [6]. The correlation map shows that SNP-scores are strongly anti-correlated between spots in the right upper corner (red in MFEA, EMCA) and left lower corner (red in WCEU, ITAP) of the map, which reflects antagonistic switching of the mean SNP-scores of the respective spots (Figure 2D, left map, red lines). About 1000 SNPs (about 10% of all SNPs tested) were collected in these two major clusters. SNP-scores between WCEU and ITAP one hand and between a series of spots characteristic for MFEA and EMCA, on the other hand, were mutually correlated, indicating closer similarity (blue lines). Spot implication analysis complements this picture by identifying spots appearing together in the individual portraits of the cultivars. Implications are visualized in the map by connecting such joint spot appearance by lines. Again, we find three major patterns, namely co-occurrence of spots in the left, the upper and the right part of the map, respectively. Interestingly, implication and correlations links along the lower border of the map are virtually absent, which means that spots from WCEU and IBER virtually don’t co-occur and are not correlated. This lack of links suggests a barrier for genomic exchange between these regions. In summary, SOM portraits visualize the genomic diversity of grapevine cultivars with single-accession and regional resolution. Overall, we identified the characteristic differences of genomic features (SNP-profiles, variance, implications and correlations) between WCEU and ITAP, IBER and cultivars from more eastern regions ranging from BALK to EMCA, MFEA and NEWO.

### 3.3. SNP Distributions and Associations with Vine Utilization

Next, we analyzed the distribution of selected SNPs across the map. Laucou et al. [11] identified 14 SNPs that allow one to assign the 783 cultivars studied. These SNPs are found mostly in areas of our map, which are attributed to red positive eMAF regions observed for WCEU/ITAP and IBER geographic regions (Figure S1A in Additional File 4: Supplementary Figures). Hence, cultivars were identified using SNP-markers, showing increased eMAF values in European cultivars and decreased values in Eastern ones. Interestingly, the number of these SNP-markers roughly agrees with the number of ‘spot-modules’ of correlated SNPs found in our SOM-analysis. Hence, our data-driven dimension reduction of feature space confirms previous results, namely, that about one-two dozen single SNPs’s are sufficient to describe roughly the genetic diversity of vine cultivars [11]. Spot clusters contain typically a few hundreds of correlated SNPs that make them robust fingerprint markers of cultivar diversity. Moreover, our SNP portrayals clearly show that more subtle and more diverse SNP patterns are hidden beyond this first, relatively coarse level of genetic diversity. In principle, each accession is genetically unique with possible impacts for diverse phenotypic traits and associated genetic patterns. Their discovery requires more extended genetic characterization, e.g., by means of sequencing approaches, and more detailed genomewide association data. Our SNP-portrayal provides an option to deal with such increased data in future applications.

The chromosome-wise distribution of SNPs shows local enrichment in most cases (see the distribution of SNPs from Chr. 1 and 2 in Figure S1B in Additional File 4 as examples), however, overall there is no clear relation between the distribution of SNPs along the genome and their location in the map. Mapping of SNPs from the different spots along the chromosomes shows a similar result, namely that overall there is no clear one-to-one relation between distribution along the chromosomes and across the map, despite the local accumulation of spot-SNPs on different chromosomes (examples for six spots are shown in Figure S1C, Additional File 4).

**Figure 3:**
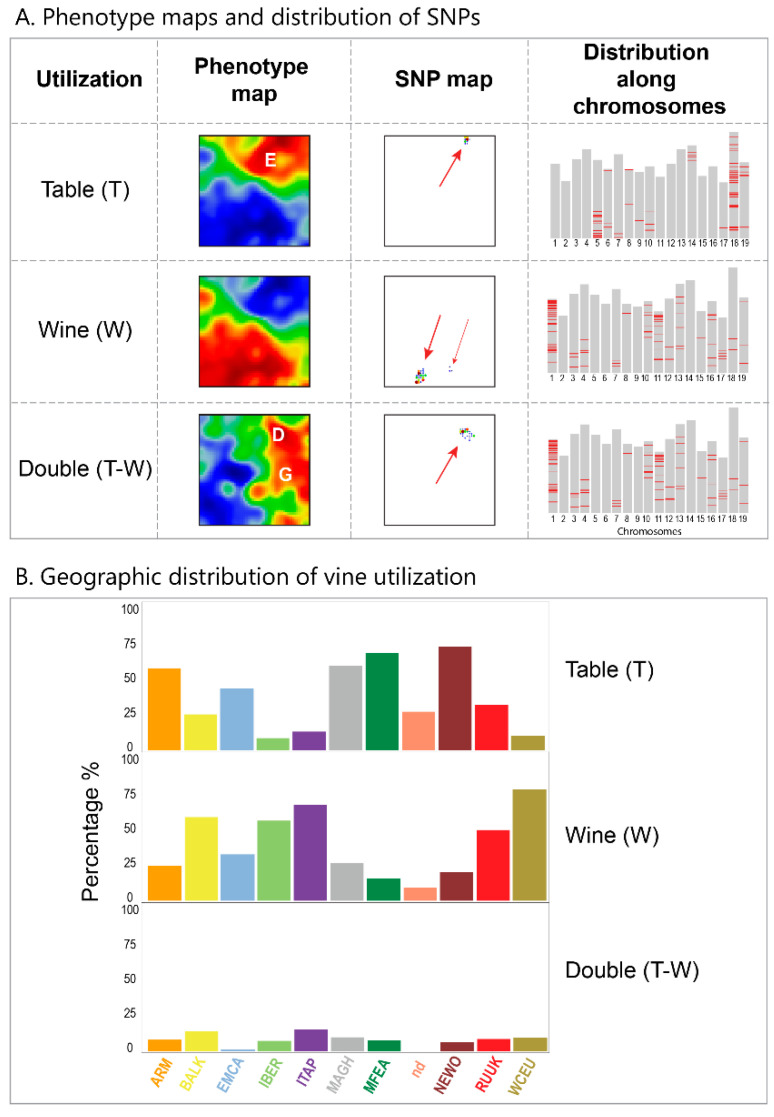
Association with vine utilization: (**A**) Phenotype maps color code correlation between SNP-scores and cultivar-utilization (table, wine, double) between red (high correlation) and blue (low correlation). Top 100 correlated SNPs accumulate in narrow regions of the SNP map (see arrows) in and around spots E (table utilization), H (wine) and D (double) and on selected chromosomes (assembly version 12X.V2). (**B**) Distribution of vine utilization (percentage of the respective vine cultivars) indicates enrichment of table utilization (T) in Eastern cultivars, of wine utilization (W) in western ones (WCEU, ITAP, IBER, BALK), while the distribution of double usage cultivars partly resembles that of wine vines however with increased frequencies in MFEA and MAGH.

Phenotype maps visualize the correlation between the SNP-scores and the utilization for the table, wine or double usage (Figure 3A). We find strong correlations (Pearson’s correlation coefficients r = 0.7) for table usage in the right upper corner of the maps, while wine usage correlates with SNPs enriched in the lower-left corner. Correlation with double usage cultivars is smaller (r = 0.3) and refers to SNPs accumulation around spot D. The top hundred SNPs with the largest correlation coefficients localize in narrow areas in the genetic landscape. They also accumulate in different chromosomal regions (Figure 3A).

**Figure 4:**
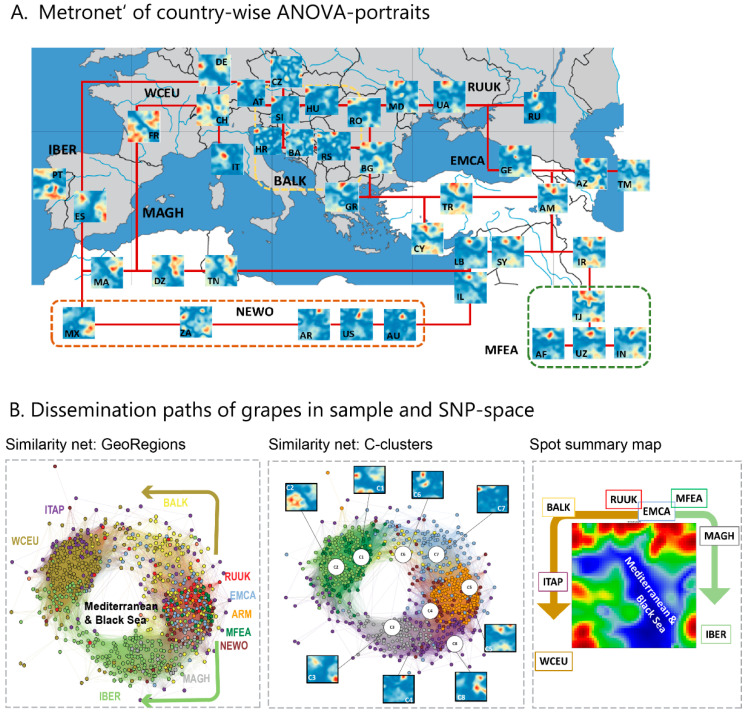
Distribution of grapevines around the Mediterranean and Black Sea: (**A**) Country-wise ANOVA portraits of cultivars are linked by a ‘metro-net’, which visualizes the relatedness between similar portraits in neighboring countries. (**B**) The network of mutual correlations between the SNP-portraits arranges them in a ring-shaped fashion, resembling the ordering of geographic regions around the Mediterranean and Black Sea. Moreover, the activated spots in the SOM order roughly in this way. Recoloring of the cultivars using the eight ‘C’-clusters taken from [11] sorts them in a consecutive way.

The phenotype map reflects the preferential utilization of cultivars used for table consumption in the East, while wine utilization dominates in Europe (WCEU, ITAP, BALK, IBER). Overall, we find an association between vine utilization and specific areas of the SOM, which reflects an East-to-West gradient with preferential table usage in the East and wine usage in the West in agreement with [8]. Note that these two spot-areas of the table- and wine-vine enrichment constitute the major ‘axis’ of anticorrelated SNP-patterns (compare with correlation net in Figure 2D), which, in turn, refers to the east-to-west ‘gradient’ of vine utilization established previously [10]. Interestingly, grapes for double wine and table usage refer to a region of the map linking MAGH and IBER cultivars, i.e., a region merging Islamic and Christian traditions (see [10] and Discussion). Another ‘transition’ region are BALK and ITAP, where however wine and table grapes are used in parallel. Additionally, the frequency distributions of cultivars, according to their utilization, supports the East-West gradient (Figure 4C). The table utilization of grapes dominates in the east and in MAGH while wine utilization dominates in Europe, where the region between MAGH and IBER forms a transition range enriching grape of double wine and table usage.

### 3.4. Genetic Relatedness of Cultivars around the Mediterranean Sea

To increase geographic resolution, we calculated country-wise SNP-landscapes using ANOVA (analysis of variance)-portrayal (Figure 4A). The ANOVA-portraits show regions of the large variance of the SNP-score in red and of low variance in blue. Inspection of the country-related portraits reveals overall a high diversity, where, however, portraits of countries from the same region often resemble each other. Moreover, the textures alter in a systematic way between the regions, e.g., from the east (EMCA, MFEA) to the west (via RUUK, BALK to WCEU, ITAP and IBER), as visualized by the ‘metro-net’ lines linking similar country portraits. Accordingly, one finds similarities of these portraits for neighboring countries from Georgia, via Russia, Ukraine and Moldova, towards Balkan countries into the west direction and from Georgia and Armenia via Iran towards Tadjikistan, Uzbekistan and Afghanistan, into the Middle Asia region (MFEA). Another route is directed from the Caucasus via Lebanon, Israel towards North Africa (MAGH) and Iberian Peninsula (IBER). The South Caucasus is also linked via Anatolia (Turkey), Cyprus with the Balkan. In the western part of Europe, portraits from Spain show similarities with Northern African countries (MAGH), and only partly with French and German portraits, which, in turn, show similarity links via Switzerland, Austria and the Czech Republic towards Balkan. Mexican cultivars resemble Spanish ones according to their SNP-portraits while cultivars from USA, Australia and Argentina, on average, reflect more similarities with grapevines from MFEA and EMCA.

In order to evaluate the relatedness between the individual SNP-portraits of cultivars, we calculated similarity nets. They are based on Pearson′s-correlation coefficients between the individual SOM-portraits and arrange them in a way that highly correlated cultivars were connected by lines and locate in the close mutual distance (Figure 4B, left). The net divides into the three major clusters enriched in cultivars from WCEU (and ITAP), MFEA and IBER, respectively, which were separated by regions of decreased accession density, especially between the WCEU and IBER clusters, reflecting reduced numbers of correlation links. The spot summary map (Figure 4B, right part) indicates exactly the same properties in SNP-feature space: the blue, low eMAF region separates spots of high eMAF-values observed in WCEU, IBER and the ‘East’. Importantly, these regions group around the Mediterranean and Black Seas, suggesting more close genetic relatedness among the cultivars along a northern and a southern coast-route, respectively (see arrows in Figure 4B). Besides the clear enrichment of cultivars from different geographic regions in the clusters, one also finds the considerable intermixing of them, reflecting genetic exchanges between the regions (see next subsection). The stratification of accessions into eight clusters (C1–C8), as proposed previously [11], provides a better ‘resolution of the correlation structure of cultivars in the similarity net (Figure 4B, C-clusters). Hence, ‘country-wise’ SNP-(ANOVA)-portrayal provides an intermediate level of resolution in-between the geographic regions and the individual cultivar portraits. It enabled us to establish their mutual relatedness, and to identify similarity links between them, ranging from the Middle East and the Caucasus to Iberia. Main roads of relatedness between the SNP-portraits of grapevine cultivars are found along a northern and a southern route about the Mediterranean and the Black Sea. A marked genetic gap is found between IBER and WCEU, suggesting that the Pyrenees are the main genetic barrier in the West. ‘Eastern’ cultivars, especially from the MFEA and EMCA regions, form a relatively tight cluster of mutually similar genomes. It links to the western cultivars via BALK and MAGH, along the northern and southern routes, respectively.

### 3.5. Diversity Trees Suggest South Caucasus as a Crossroad of Grapevine Dissemination

**Figure 5:**
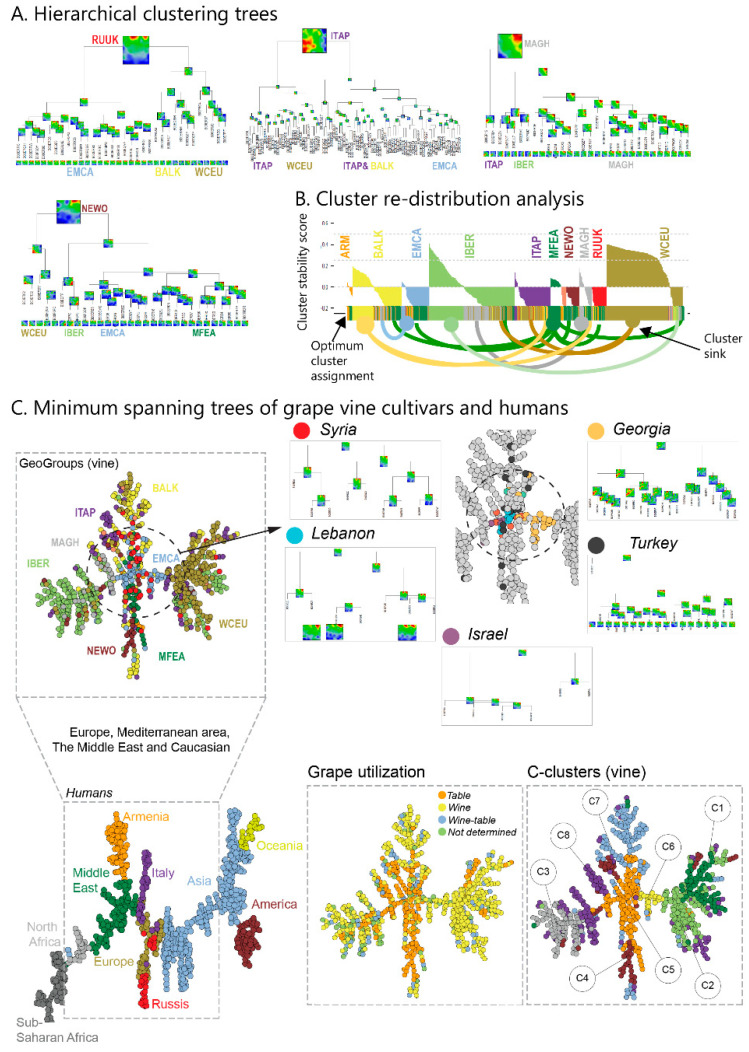
Genome diversity in and between the geographic regions: (**A**) Hierarchical clustering of vine cultivars from selected regions shows overlapping genomic properties. (**B**) The cluster stability plot (‘silhouette plot’) indicates intra- and inter-cluster similarities. The similarity score is positive for stable cluster-assignment of cultivars and negative, if a cultivar better matches into another cluster (the best-matching cluster for each cultivar is indicated by the colored bar below the silhouette plot). The arc-lines link unfavorable cluster assignments with the respective sink (best matching)-cluster. (**C**) The minimum spanning tree (MST) of the vine cultivars reveals four major leafs enriching IBER (and MAGH), BALK (and ITAP), WCEUR and MFEAS (and NEWO) grapes, respectively. The central ‘cross-road’ linking the four leafs enriches EMCA-cultivars. Particularly, cultivars from Lebanon, Israel and Syria locate within the ‘cross-road’ region (see an enlargement in the right part). Vines from Georgia form the trunk of the European part. Armenian and Turkish vines spread more widely along the MST. Re-coloring the MST according to grape utilization shows that table usage predominates along the vertical trunk of the MST (MCEU, EMCA) and along the MAGH-branch, while wine usage is found mainly for European grapes. Alternative clustering, as proposed previously (C-clusters) [11], better separates the clusters along the MST. The MST of human genomes of modern populations from Africa to Oceania taken from the Human Genome Project (HGP) shows a more linear arrangement (left part), reflecting different dissemination patterns. The dashed rectangle includes populations of the same geographic regions, as studied for the grapevine cultivars.

In the next step, we analyzed SNP-portraits and their diversity on the level of individual cultivars. An inspection of their gallery (Additional File 1) indicates typical patterns for their geographic regions, which, however, partly mix between them. Hierarchical clustering trees of cultivar portraits selected from four different regions illustrate this mixing effect (Figure 5A). For example, cultivars from RUUK split into groups resembling genomic patterns from EMCA, BALK and WCEU, respectively. Cultivars from MAGH divide into groups resembling IBER and ITAP patterns, but also a larger group of ‘local’, MAGH-like ones. To quantify this intermixing, we applied re-distribution analysis (Figure 5B), which uses a cluster stability score to estimate whether a certain accession better fits into its geographic region (positive score value) or into another one (negative score value). MFEA cultivars show high intra-cluster stability, followed by WCEU, BALK and MAGH, while, e.g., RUUK cultivars tend to distribute virtually completely into other clusters. Hence, the MFEA region can be interpreted as a sink for cultivars from other geographic regions, except for ITAP and, partly, WCEU. EMCA genomes are similar to MEFA genomes, as indicated by the fact that the latter ones (including ARM) nearly exclusively tend to shift into the cluster of the former one.

To further evaluate the relatedness between the cultivars, we calculated a minimum spanning tree (MST), which, in contrast to the correlation net discussed above, connects only most similar cultivars and, in consequence, generates ‘paths’ of closest similarities (Figure 5C). The MST obtained reveals a cross-like shape, showing four major branches. Cultivars from IBER (left branch), WCEU (right branch), BALK and ITAP (top) and MEFA (down) accumulate in the four major branches, while EMCA cultivars are found predominantly in the central crossroad area (Figure 5C). The ‘cross-like’ structure of grapevine cultivars suggests their distribution along four paths with their common origin in the EMCA area. Zooming in reveals that Georgian cultivars form the trunk of the WCEU-branch to the right, while cultivars from Syria, Lebanon and Israel are at the origin of the trunk to the left IBER and MAGH branch. Notably, Georgian accessions split in portraits more similar to western Europeans, to Greek ones and to typical EMCA portraits (see the hierarchical clustering tree of Georgian vines in Figure 5C), which reflects a large variability of their genomes. Armenian and Turkish cultivars distribute more in the vertical direction along the MFEA and BALK branches. Coloring of the MST according to grape utilization shows strong enrichment of table cultivars along the vertical trunk enriching MFEA grapes, while wine cultivars enrich in the European vine branches referring to WCEU, IBER and BALK. Alternative recoloring the MST using the alternative ‘C‘-clustering [11] better adjusts to the cross-like structure, where C5 collects the cultivars from the crossroad area, except the Georgian ones (C6) (Figure 5C). Interestingly, WCEU cultivars split into a C1 and C2 clusters with C1 collecting cultivars from Germany and Austria, while C2 enriches cultivars from Italy.

Note, that the structure of the vine-MST is completely different from the MST of human genomes of worldwide populations taken from the human genome project. This MST of human genomes possesses an almost linear backbone, with only a few side branches. It reflects the ‘out of Africa’ basic migration history of modern humans, which initially proceeded mostly along one major path. Hence, MST presentation seems to reflect the mechanisms and paths of genome dissemination. In summary, diversity analysis on single cultivar level reveals a marked intermixing between the different geographic regions where, however, cultivars from the South-Caucasus and Near East from the trunk for diverging branches of vines are typical for IBER (and ITAP), WCEU, MFEA and BALK (and RUUK) regions, respectively.

### 3.6. ‘Pseudotime’ Suggests Different Scales of Cultivar Diversification

**Figure 6:**
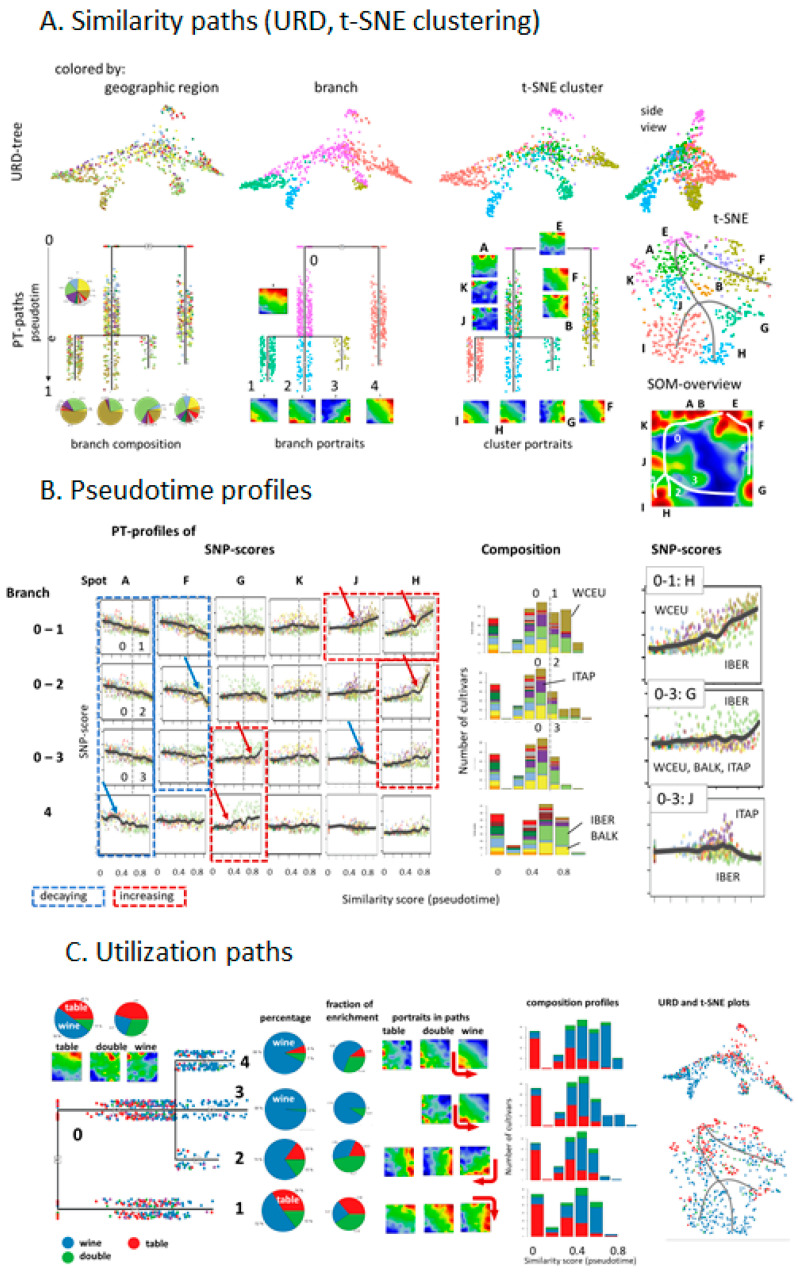
Multibranched pseudotime analysis: (**A**) Similarity paths using URD [26] reveal a ‘Kraken’ like distribution of vine cultivars which is described by four ‘tentacles’ as pseudotime branches. They accumulate cultivars preferentially from WCEU and IBER (see pie diagrams), respectively. t-SNE clustering provides ten clusters labelled by the most prominent SNP-spot expressed in each of them (right part). The grey and white curves visualize the paths of cultivar similarities in the t-SNE accession- and SOM SNP-space, respectively. (**B**) Pseudotime analysis reveals increasing (marked by red dashed rectangles) and decaying (blue rectangles) courses, indicating, e.g., accumulation or depletion of WCEU cultivar features and accessions at later PT values. Arrows indicate points of increasing (red) and decreasing (blue) slopes. Composition of the geographic origin of the cultivars is shown as barplot (middle). Enlarged SNP-profiles, as shown in the right part of the figure, reveal details of cultivar distribution around the LOESS-fits (locally weighted scatterplot smoothing, black curves). (**C**) The utilization of grapes (wine, table, double wine and table) along the developmental paths indicates that wine usage appeared at later PT and along all branches compared with table utilization. The pie diagrams show percentages of grape utilization along the branches (larger diagrams) and relative percentages (smaller pie diagrams) as ratio percentage along the branch divided by the overall percentage (wine: 61%, table: 25%, double: 12%). The mean SOM-portraits refer to cultivars of different utilization along the branches. SNP-spots shift into the direction as indicated by the arrows.

For a more detailed study of possible paths of the distribution of grapevine, we applied so-called ‘pseudotime’ (PT) analysis. It arranges cultivars according to their mutual genetic similarity along possible multi-branched paths, using a diffusion-law based algorithm [26]. The obtained paths thus describe the dissemination of grapevine cultivars between the geographic regions, assuming a starting area which we set to cultivars from the ‘cross-road’ part of the MST (see the previous section). PT-analysis assigns a similarity measure called ‘pseudotime’, normalized between zero (start) and unity (end). The grapevine accessions sort into four branches, where each of them collects cultivars of mixed geographic origin (Figure 6A, left part). At the root the tree divides into two branches (numbered ‘0′ and ‘4′), where ‘0′ collects predominantly MFEA, BALK and ITAP cultivars and ‘4′ enriches grapes from IBER and MAGH. Branch ‘0′ further splits at larger PTs into three sub-branches (‘0–1′ to ‘0–3′) accumulating WCEU, ITAP and again IBER vines, respectively. Mean SOM-portraits along the branches reveal clear differences in the SNP-landscapes, supporting their assignment to BALK and RUUK (branch 0), WCEU and ITAP (0–1), WCEU (0–2), IBER (0–3) and MFEA and EMCA (4) characteristics (Figure 6A, middle column). Moreover, t-SNE clustering provides a more diverse stratification of cultivars into ten groups, where most of them were characterized by one-to-two major spots in their portraits (Figure 6A, right part). Importantly, spots appear in a consecutive fashion along the paths; this way providing developmental traces in the SNP-landscape as illustrated by the white curves in the SOM-overview map (Figure 6A, right part).

The consecutive alterations in feature space observed were supported by PT-profiles of selected spots (Figure 6B). The eMAF SNP-score of spot A (up in EMCA) decays along all branches (blue, dashed frames), reflecting increasing genetic distance from this region with increasing PT. In contrast, spot F (up in MFEA and MAGH) remains relatively invariant along branch 4. The IBER and WCEU related spots gain in ‘later’ PT along different paths (red dashed frames), namely, path 4, 0–1 and 0–2, respectively. These courses, however, differ in their turning points where eMAF starts to increase and in their slopes. For example, the gain of 0–2 starts at later PTs and it is steeper compared with 0–1, suggesting different ‘switching points’ and time-scales of grapevine distribution (see red arrows). Interestingly, ITAP-cultivars accumulate at intermediate PTs along the spot profile J of all subbranches 0–1, –2 and –3. Bar plots of the composition of cultivars along the branches, as a function of PT, support the accumulation of MFEA, EMCA, RUUK, MAGH, and partly ITAP vines at early and intermediate PTs while WCEU and IBER (and partly BALK along path 4) cultivars accumulate at later PTs. Hence, PT-scaling obviously quantifies aspects of dissemination of cultivars in East-to-West direction via different routes.

The consideration of grape utilization along the different PT-paths clearly reveals the enrichment of table vines in branches 0 and 4, while wine cultivars strongly enrich in the branches 1–3 at later PTs (Figure 6C, pie diagrams). The gain of wine usage also becomes evident in the PT-profiles at PT > 0.5, and, on the other hand, table vine enrichment at PT < 0.5 (Figure 6C, bar plot). Stratification of the portraits along the paths reflects the systematic shift of spot patterns between the portraits of the table and wine vines from the upper to the lower parts of the maps along the paths shown in Figure 6A (see also the arrows in Figure 6C, middle). The URD- and t-SNR plots support this result by the separation between table (red color) and wine (blue) cultivars in top-to-down direction (right part in Figure 6C).

In summary, multibranched PT-analysis resolves the ‘transverse’ distribution of cultivars among geographic regions also in ‘longitudinal’ PT-dimension, by sorting them according to mutual similarities of their genomic landscapes. We find the accumulation of grapes used for wine production at later PTs especially in Europe, which suggests the diversification of wine cultivars at later times compared with table grapes.

## 4. Discussion

**Figure 7:**
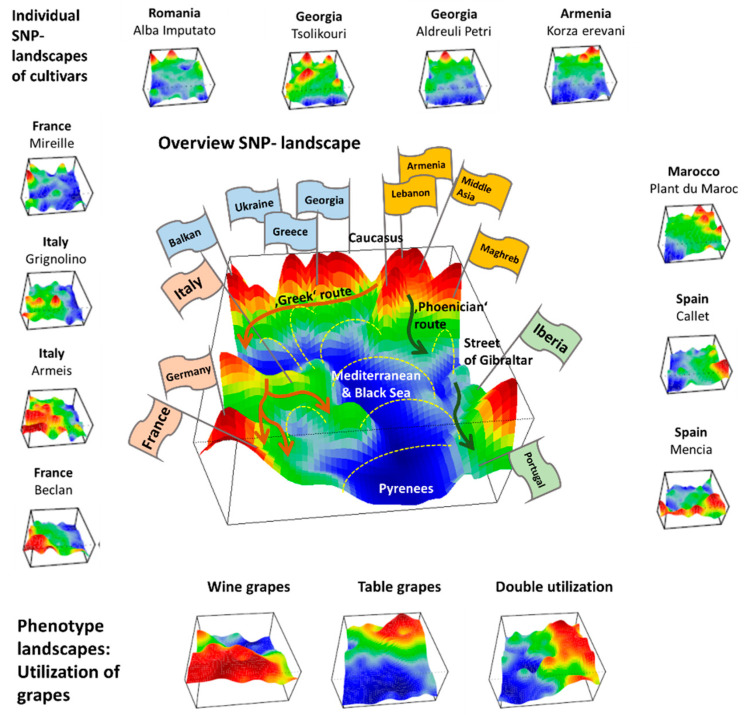
Genomic landscapes of grapevine cultivars: The SNP-landscapes of all 783 cultivars (examples from different regions are shown as small images) were summarized into a mean ‘overview’ landscape (large image with flags). Correlated SNP-profile self-organized together due to Figure 7 examples are shown as small images). The ‘portraits’ project the multidimensional similarity space spanned by the excess minor allele frequencies (eMAF) of the 10,207 SNPs under study into two dimensions. The vertical ‘height’ dimension visualizes the eMAF-value of the SNPs defined as the deviation of its minor allele frequency (MAF) in a certain cultivar from its mean MAF-value averaged over all cultivars. The algorithm ‘self-organizes’ similar SNP-profiles more closely together than different ones. In the result, we obtained 783 different SNP landscapes, each showing a unique combination of red ‘mountain-ridges’ and blue ‘valleys’ referring to positive and negative eMAF-values, respectively. The overview landscape summarizes the diversity of the individual portraits in terms of frequently observed SNP-patterns (Figure 7, large image). Its topology reveals several ‘mountain areas’ which refer to cultivars from different geographic regions. It relates to the geographic origin of grape cultivars, their phenotypes and history of distribution throughout the ‘classical’ world, starting from its origin of domestication about 10,000 years ago. Particularly, the genomic landscape resembles the geographic map around the Mediterranean and the Black Sea. Namely, the eMAF-‘mountains’ order cultivars from the Caucasus along a northern route’ via Balkan towards Western Europe and along a southern route via Palestine and Maghreb towards the Iberian Peninsula. A central ‘blue valley’ referring to predominantly negative eMAF-values separates both routes. It can be interpreted geographically as the Mediterranean and Black Sea areas, which obviously constitute areas of reduced genetic exchange. Interestingly, the largest barrier is found between grapes from the Iberian Peninsula and Western Europe (France, Italy), while the street of Gibraltar appears only as a small sidearm of the central ‘genetic’ valley, thus indicating a relatively moderate genetic barrier between North Africa and Iberia. Hence, Iberian Peninsula and northern Africa could be considered subcontinents of Europe, being separated by the Pyrenees according to vine genetics, which also agrees with the recent comparison of human genomes from these areas [28]. Another moderate genetic barrier is found between grapes from the Balkan and Western Europe (Germany, Switzerland and Italy). According to these barriers, cultivars divide into three major groups on the coarsest level of classification, namely Western Europe and Italian grapes, Iberian grapes and vine cultivars from Eastern and Maghreb regions. Detailed inspection of the mountain range of ‘eastern’ grapes reveals fine internal structure of valleys separating, e.g., Armenian from Georgian grapes and vines from Anatolia and Greece from Balkan ones.

We applied SOM artificial neural network-based machine learning to genome-wide SNP data of 783 grapevine cultivars, in order to visualize and to analyze the landscape of grapevine genomes, in terms of topological features such as ‘mountains’ and ‘valleys’, referring to positive and negative eMAF values, respectively (Figure 7).

We complemented this genetic feature (SNP-) landscape by grapevine accession-similarity plots. Each of them visualizes different aspects of the mutual relatedness of cultivars. Principal component plots distribute accessions in (eMAF) distance-scale. It results in local overcrowding of cultivars with similar genomes, e.g., from Near East, Caucasus and Middle Asia while Western European and Iberian cultivars separate into virtually different data clouds of large variability between sample genomes. PCA requires separate projection-plots to cover the essential properties of similarity space. The (correlation) similarity net projects multidimensional sample space into two dimensions. It reveals major routes of grape distribution around the Mediterranean Sea and the genetic barrier between Iberian and Western European cultivars, evident also in the feature landscape. In contrast, the minimum spanning tree (MST) generates path-like structures. For worldwide human genomes, the MST reflects roughly the ‘out-of-Africa’ migration history, in terms of a virtually linear path starting from Sub-Saharan Africa and ending in East Asia, with side branches towards Europe, America and Oceania. Interestingly, the grapevine MST splits into four mean branches, enriching West European, Iberian, Balkan and Middle East grapes, and with cultivars from South Caucasus and Fertile Crescent (Syria, Lebanon, East Anatolia) at their central crossroad. A detailed comparison of individual (ANOVA-) portraits further supports this result, using the feature landscapes in a country-wise resolution.

Overall, our genomic landscape and the different sample similarity plots are consistent with the historical knowledge and previous genetic findings of grapevine domestication and distribution [7,10]. Accordingly, cultivated grapes occurred initially in South Caucasus (Georgia, Armenia) and Fertile Crescent (East Anatolia and North Lebanon and Syria) [29,30]. Indeed, grapes from this region form the crossroad in the MST, presumably due to footprints of initial cultivation in their genomes. Grapes then distributed towards the ‘classical’, Mediterranean world into the west direction and into East towards Iran and the Middle East (Tadjikistan, Uzbekistan), Afghanistan and India. The northern and southern ways into the West agree with the distribution of settlements of Greeks (the Black Sea, including Crimea, Anatolia, Southern Italy and Sicily, Southern France and Northern Spain along the Northern coast of Mediterranean Sea) and Phoenicians (Lebanon, Carthago/Tunis, Maghreb and South-West Spain), respectively. Genomes of vines from the Italian Peninsula are very diverse and reflect links to almost all parts of the Mediterranean area (BALK, EMCA, MAGH, IBER) and Western Europe, presumably due to intense cultural exchange in the Greek world, and later within the Roman empire [31].

Hence, grape growing and winemaking in classical time disseminated from ‘Iberia-to-Iberia’, starting in or near the ancient kingdom Iberia in Middle Georgia in South Caucasus in the East and ending at the Iberian Peninsula in the west. The combined action of selection, breeding, admixture and migration have shaped the cultivated grape diversity. Substantial genetic diversity has been maintained, subsequent to domestication derived from Transcaucasian Wild Grape, possibly due to several events of introgression from local wild vine Silvestris-varieties, Wild grapes (*V. vinifera* ssp. sylvestris), particularly in Western Europe [7]. Although Mediterranean and Black Seas served rather as highways of cultural exchange than as barriers, vine distribution obviously followed primarily ‘country ways’ along with the coastal areas.

We visualize grape utilization in terms of phenotype maps which associate table, wine and double usage with different geographic regions (Figure 7, part below). Grapes for fresh consumption (table vines) predominate in Eastern and North African areas, while wine utilization is found mostly in Western Europe (RUUK, BALK, WCEU, ITAP, IBER). This division has been associated with different religious rules concerning alcohol consumption in Islamic and Christian regions [8]. Interestingly, the red region of wine-utilization covers the WCEU and IBER geographic regions, and thus it bridges the genetic border formed by the Pyrenees in the overview landscape (Figure 7).

In order to extract additional information about the dissemination of cultivars, we applied pseudo-time (PT) analysis. This method has been originally developed for describing cell differentiation using single-cell transcriptomic data [26,32], and it was applied to study human cancer progression using single-cell and bulk transcriptome data [14,33]. In the context of this study, PT sorts and scales vine genomes according to their genetic similarity, into a directed, branched graph. Starting with the assumption of initial Eastern domestication of the grapevine PT then describes its dissemination in an East to West direction. Vines from the Caucasus and the East refer to small PT-values and vines from the Balkan and Italian peninsula mostly to intermediate PTs. Interestingly, the genetic characteristics of Western European and Iberian grapes follow different courses at later PTs along different branches, which eventually reflects different waves of cultivation, since the Roman Empire times. Particularly, recent pedigree analyses uncover a putative Middle Age cultivar melting pot, giving rise to many of today’s cultivars, suggesting even secondary domestication events taking place in Western Europe and the Iberian Peninsula ending in the cultivars that are used in viticulture today [8]. Different PT-branches show different slopes and switching points in their grow regimes at later PTs. Table grapes mostly accumulate at early PTs. Hence, our ‘SOMelier’ transforms vine genomes into a landscape resembling the topology of the geographic regions of grape cultivation and/or the diversity of their genomes. Different methods of similarity analyses in SNP-feature and cultivar space enable extracting details of dissemination history and the utilization of grapes.

## 5. Conclusions

Our ‘SOMmelier’ approach visualizes essential aspects of grapevine genomes related to geographic distribution, paths of dissemination and vine utilization. ‘SOMmelier’ portrays vine genomes in terms of individualized images. They are intuitive, meaning that they don’t need specialized genetic knowledge for interpretation. Together with vine genome landscapes described here, we propose to use individual genomic portraits as an option to supplement vine cultivar passports as fingerprint characteristics of their genomes. Such fingerprint portraits consider virtually the whole diversity of the vine. Note also that we here discuss accession portraits mainly on the level of geographic regions as a sort of worked example to support the interpretation of genomes of individual accession portraits. Our study also demonstrates that bioinformatics methods proven before in analytic tasks on different omics realms, mostly transcriptomics, but also epigenomics, proteomics and human genomics, provide reasonable results if applied to vine cultivars as an example of plant genomes. Our approach thus extends the methods toolbox for plant genetics by providing novel approaches which complement established ones. Their pros and cons should be evaluated in future applications. The ‘SOMelier ‘method opens the opportunity to process larger genotype data, obtained by, e.g., whole genome sequencing and/or increased number of cultivars included.

## Supplementary Materials

The following are available online at https://www.mdpi.com/2073-4425/11/7/817/s1 Additional File 1: Individual cultivar accession portraits (PDF-format). Samples are assigned according to sample IDs taken from Loucou et al. [11]. Additional File 2: The list of SNPs collected in all spots. Each sheet includes the list of SNPs for all SOM spots (A - O) (XLS-format). Additional File 3: Summary annotation of SNPs, used in this study [11] (CNV-format). Additional File 4: Additional figures Figure S1 and Figure S2 (PDF-format): Figure S1: SNP maps, eMAF profiles and associations with selected phenotypes. (a) 14 SNPs (assembly version 12X.V0) selected in [11] to identify the 783 vine cultivars accumulate in WCEU/ITAP and IBER regions of the map. Their mean SNP profile shows large eMAF-values for cultivars from these regions on the average. Profiles of selected individual SNPs reveal a nearly bi-modal distribution of eMAF-values. (b) SNPs from different chromosomes distribute over several spot regions (red frames). (c) Distribution of SNPs from selected spots (red marks) among the 19 chromosomes (grey vertical bars). Figure S2: ANOVA-portraits stratified according to geographical regions and utilization of grapes. The log P-value is visualized on the SOM map. The most significant areas for all phenotypes overlap with characteristic spots for MAGH cultivars.

## Abbreviations

**Abbreviations of counties**	
NEWO	New World
MAGH	Maghreb
EMCA	Eastern Mediterranean and Caucasus
MFEA	Middle and Far East
BALK	Balkans
RUUK	Russia and Ukraine
ARM	Armenia
IBER	Iberian Peninsula
ITAP	Italian Peninsula
WCEU	Western and Central Europe
