# Supplementary material for: SOMmelier—Intuitive Visualization of the Topology of Grapevine Genome Landscapes Using Artificial Neural Networks
Source: Genes (Basel). 2020 Jul 17;11(7):817. doi: 10.3390/genes11070817 (PMC7397337; doi:10.3390/genes11070817)
Supplement: Supplementary file 1 [file genes-11-00817-s001.zip › Additional File 1.pdf]

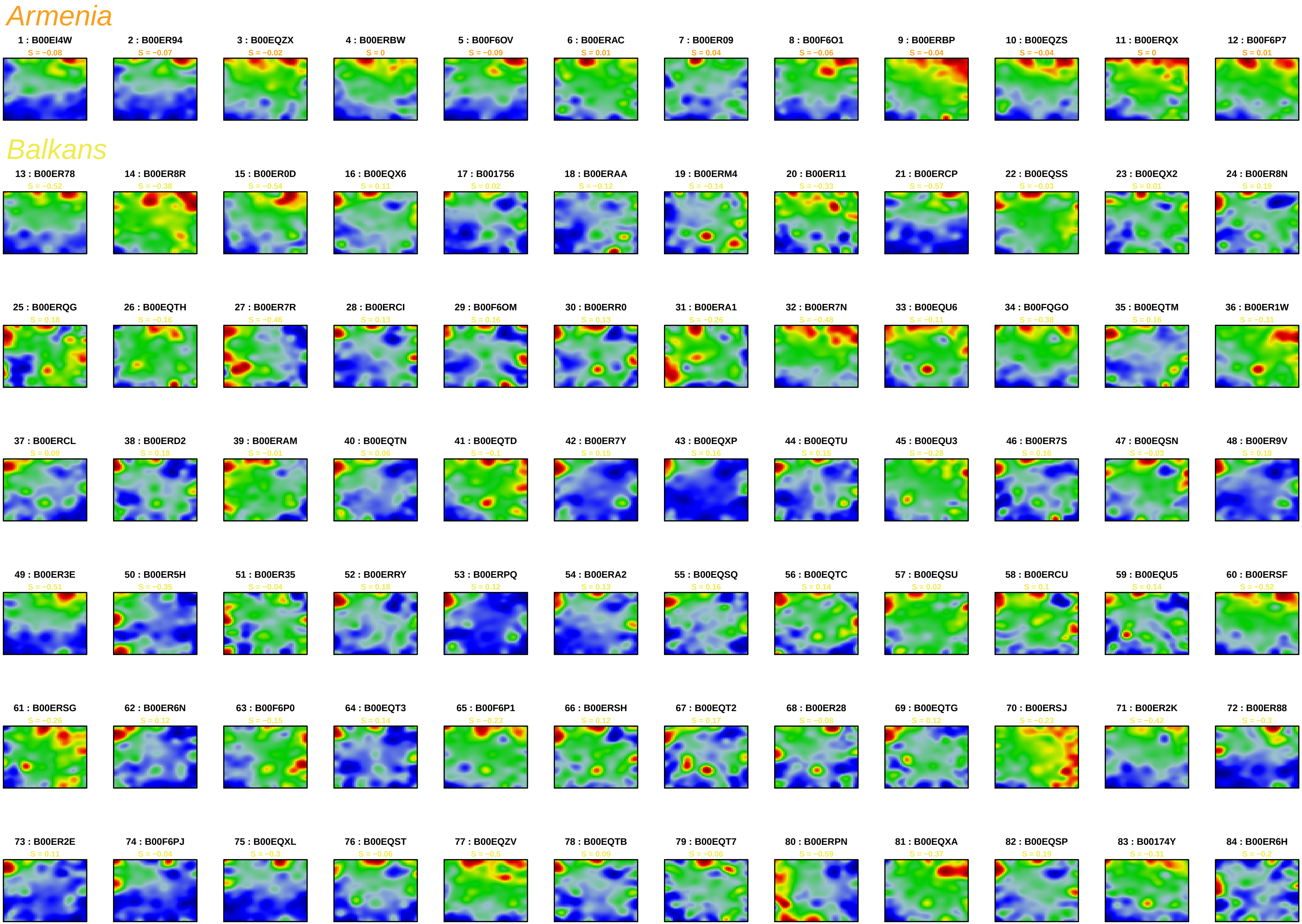
Armenia
1 : B00EI4W
S = −0.08
2 : B00ER94
S = −0.07
3 : B00EQZX
S = −0.02
4 : B00ERBW
S = 0
5 : B00F6OV
S = −0.09
6 : B00ERAC
S = 0.01
7 : B00ER09
S = 0.04
8 : B00F6O1
S = −0.06
9 : B00ERBP
S = −0.04
10 : B00EQZS
S = −0.04
11 : B00ERQX
S = 0
12 : B00F6P7
S = 0.01
Balkans
13 : B00ER78
S = −0.52
14 : B00ER8R
S = −0.38
15 : B00ER0D
S = −0.54
16 : B00EQX6
S = 0.11
17 : B001756
S = 0.02
18 : B00ERAA
S = −0.12
19 : B00ERM4
S = −0.14
20 : B00ER11
S = −0.33
21 : B00ERCP
S = −0.57
22 : B00EQSS
S = −0.03
23 : B00EQX2
S = 0.01
24 : B00ER8N
S = 0.19
25 : B00ERQG
S = 0.18
26 : B00EQTH
S = −0.16
27 : B00ER7R
S = −0.46
28 : B00ERCI
S = 0.13
29 : B00F6OM
S = 0.16
30 : B00ERR0
S = 0.13
31 : B00ERA1
S = −0.26
32 : B00ER7N
S = −0.48
33 : B00EQU6
S = −0.11
34 : B00FQGO
S = −0.38
35 : B00EQTM
S = 0.16
36 : B00ER1W
S = −0.31
37 : B00ERCL
S = 0.09
38 : B00ERD2
S = 0.18
39 : B00ERAM
S = −0.01
40 : B00EQTN
S = 0.06
41 : B00EQTD
S = −0.1
42 : B00ER7Y
S = 0.15
43 : B00EQXP
S = 0.16
44 : B00EQTU
S = 0.15
45 : B00EQU3
S = −0.28
46 : B00ER7S
S = 0.16
47 : B00EQSN
S = −0.03
48 : B00ER9V
S = 0.18
49 : B00ER3E
S = −0.51
50 : B00ER5H
S = −0.35
51 : B00ER35
S = −0.04
52 : B00ERRY
S = 0.18
53 : B00ERPQ
S = 0.12
54 : B00ERA2
S = 0.13
55 : B00EQSQ
S = 0.16
56 : B00EQTC
S = 0.14
57 : B00EQSU
S = 0.02
58 : B00ERCU
S = 0.1
59 : B00EQU5
S = 0.14
60 : B00ERSF
S = −0.52
61 : B00ERSG
S = −0.26
62 : B00ER6N
S = 0.12
63 : B00F6P0
S = −0.15
64 : B00EQT3
S = 0.14
65 : B00F6P1
S = −0.23
66 : B00ERSH
S = 0.12
67 : B00EQT2
S = 0.17
68 : B00ER28
S = −0.08
69 : B00EQTG
S = 0.12
70 : B00ERSJ
S = −0.23
71 : B00ER2K
S = −0.42
72 : B00ER88
S = −0.3
73 : B00ER2E
S = 0.11
74 : B00F6PJ
S = −0.04
75 : B00EQXL
S = −0.3
76 : B00EQST
S = −0.06
77 : B00EQZV
S = −0.5
78 : B00EQTB
S = 0.09
79 : B00EQT7
S = −0.06
80 : B00ERPN
S = −0.59
81 : B00EQXA
S = −0.37
82 : B00EQSP
S = 0.19
83 : B00174Y
S = −0.31
84 : B00ER6H
S = −0.2

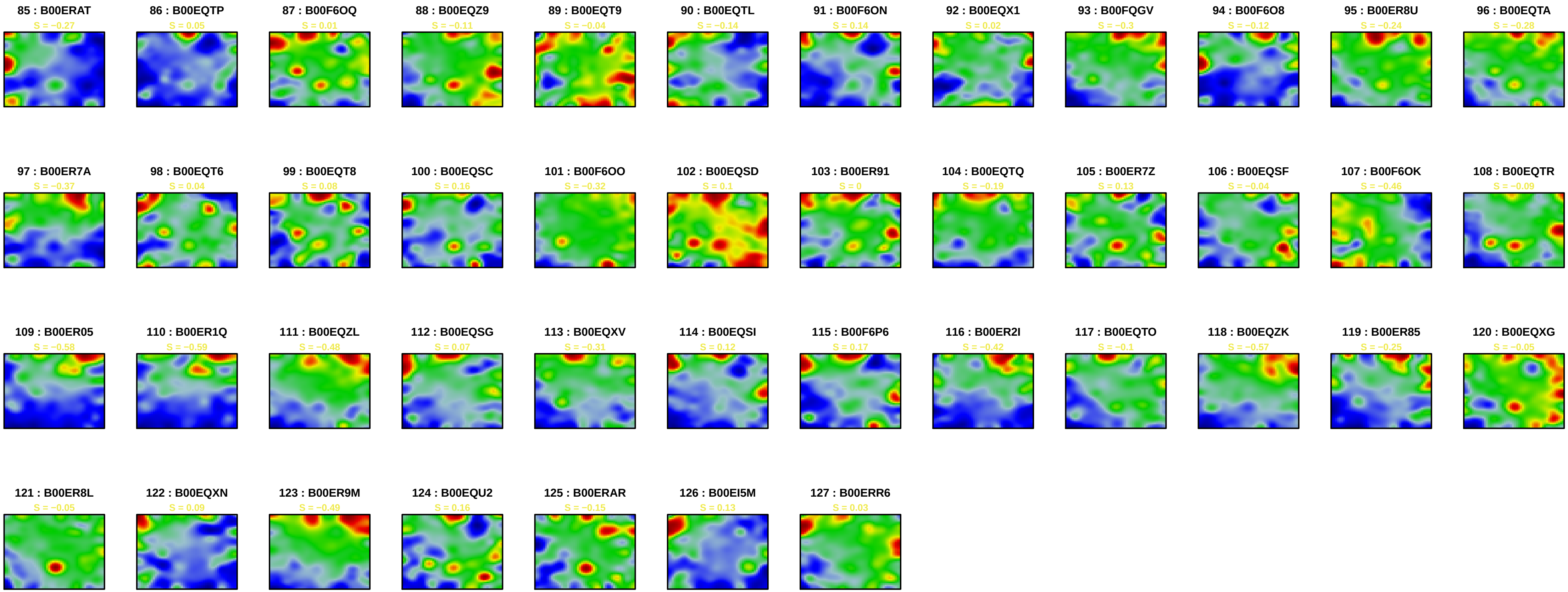

Eastern_Mediterranean_Caucasus

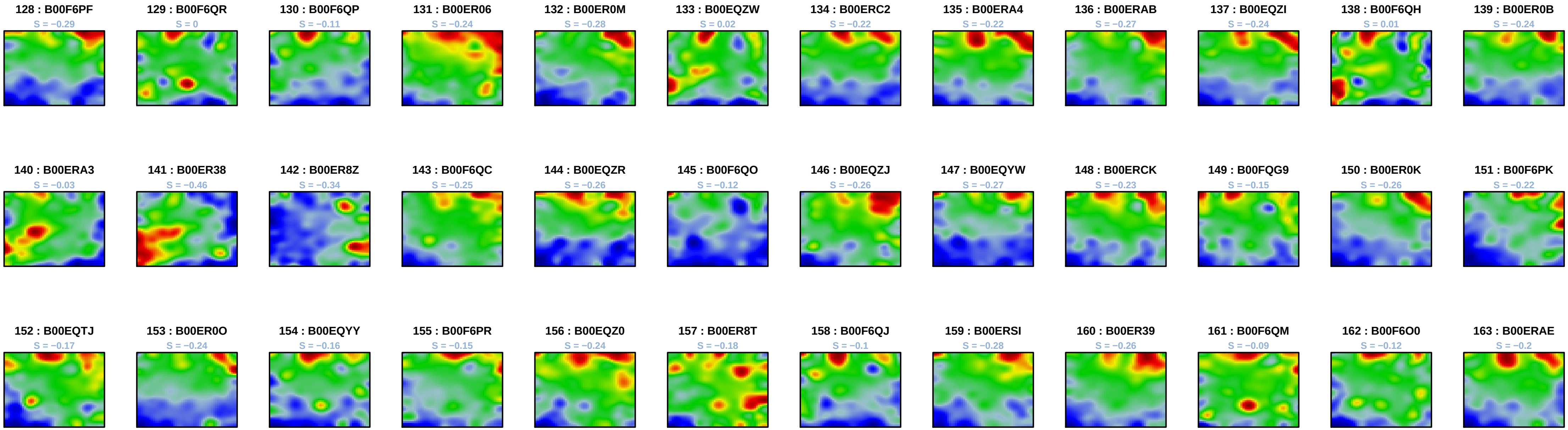

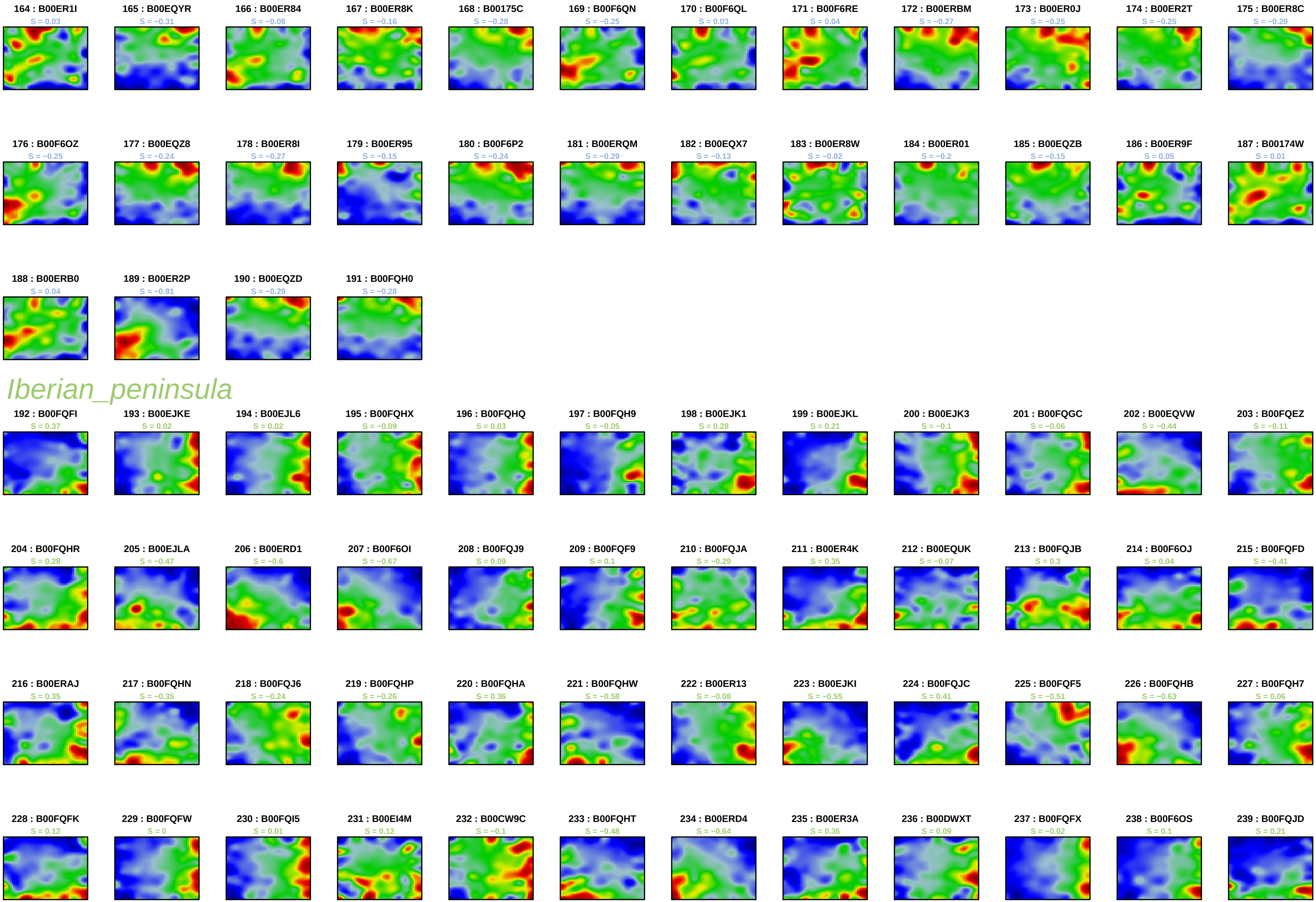
164 : B00ER1I
S = 0.03
165 : B00EQYR
S = −0.31
166 : B00ER84
S = −0.08
167 : B00ER8K
S = −0.16
168 : B00175C
S = −0.28
169 : B00F6QN
S = −0.25
170 : B00F6QL
S = 0.03
171 : B00F6RE
S = 0.04
172 : B00ERBM
S = −0.27
173 : B00ER0J
S = −0.25
174 : B00ER2T
S = −0.25
175 : B00ER8C
S = −0.29
176 : B00F6OZ
S = −0.25
177 : B00EQZ8
S = −0.24
178 : B00ER8I
S = −0.27
179 : B00ER95
S = −0.15
180 : B00F6P2
S = −0.24
181 : B00ERQM
S = −0.29
182 : B00EQX7
S = −0.13
183 : B00ER8W
S = −0.02
184 : B00ER01
S = −0.2
185 : B00EQZB
S = −0.15
186 : B00ER9F
S = 0.05
187 : B00174W
S = 0.01
188 : B00ERB0
S = 0.04
189 : B00ER2P
S = −0.81
190 : B00EQZD
S = −0.29
191 : B00FQH0
S = −0.28
Iberian_peninsula
192 : B00FQFI
S = 0.37
193 : B00EJKE
S = 0.02
194 : B00EJL6
S = 0.02
195 : B00FQHX
S = −0.09
196 : B00FQHQ
S = 0.03
197 : B00FQH9
S = −0.05
198 : B00EJK1
S = 0.28
199 : B00EJKL
S = 0.21
200 : B00EJK3
S = −0.1
201 : B00FQGC
S = −0.06
202 : B00EQVW
S = −0.44
203 : B00FQEZ
S = −0.11
204 : B00FQHR
S = 0.28
205 : B00EJLA
S = −0.47
206 : B00ERD1
S = −0.6
207 : B00F6OI
S = −0.67
208 : B00FQJ9
S = 0.09
209 : B00FQF9
S = 0.1
210 : B00FQJA
S = −0.29
211 : B00ER4K
S = 0.35
212 : B00EQUK
S = −0.07
213 : B00FQJB
S = 0.3
214 : B00F6OJ
S = 0.04
215 : B00FQFD
S = −0.41
216 : B00ERAJ
S = 0.35
217 : B00FQHN
S = −0.35
218 : B00FQJ6
S = −0.24
219 : B00FQHP
S = −0.26
220 : B00FQHA
S = 0.36
221 : B00FQHW
S = −0.58
222 : B00ER13
S = −0.08
223 : B00EJKI
S = −0.55
224 : B00FQJC
S = 0.41
225 : B00FQF5
S = −0.51
226 : B00FQHB
S = −0.63
227 : B00FQH7
S = 0.06
228 : B00FQFK
S = 0.12
229 : B00FQFW
S = 0
230 : B00FQI5
S = 0.01
231 : B00EI4M
S = 0.12
232 : B00CW9C
S = −0.1
233 : B00FQHT
S = −0.48
234 : B00ERD4
S = −0.64
235 : B00ER3A
S = 0.36
236 : B00DWXT
S = 0.09
237 : B00FQFX
S = −0.02
238 : B00F6OS
S = 0.1
239 : B00FQJD
S = 0.21

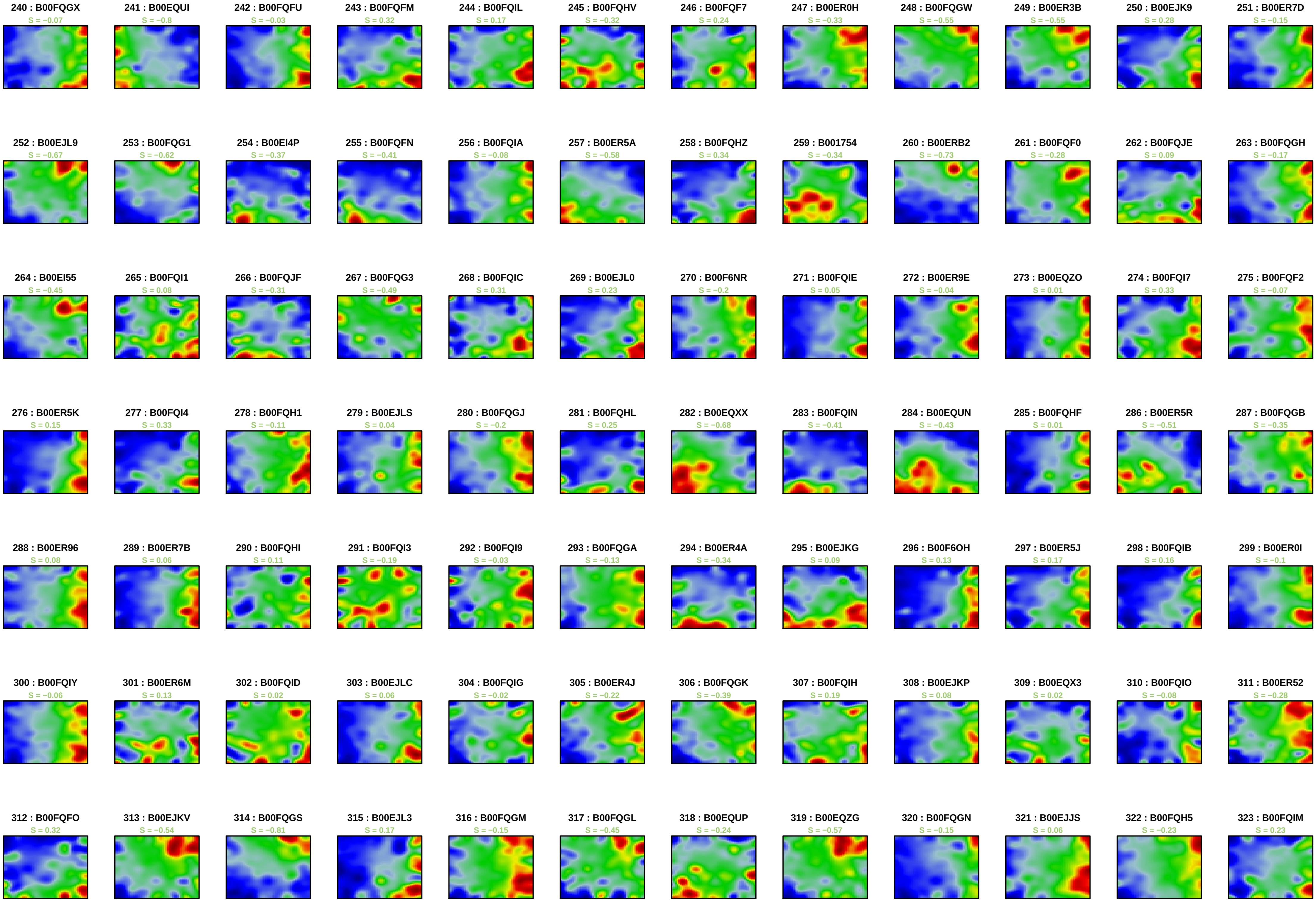

240 : B00FQGX
S = −0.07
241 : B00EQUI
S = −0.8
242 : B00FQFU
S = −0.03
243 : B00FQFM
S = 0.32
244 : B00FQIL
S = 0.17
245 : B00FQHV
S = −0.32
246 : B00FQF7
S = 0.24
247 : B00ER0H
S = −0.33
248 : B00FQGW
S = −0.55
249 : B00ER3B
S = −0.55
250 : B00EJK9
S = 0.28
251 : B00ER7D
S = −0.15
252 : B00EJL9
S = −0.67
253 : B00FQG1
S = −0.62
254 : B00EI4P
S = −0.37
255 : B00FQFN
S = −0.41
256 : B00FQIA
S = −0.08
257 : B00ER5A
S = −0.58
258 : B00FQHZ
S = 0.34
259 : B001754
S = −0.34
260 : B00ERB2
S = −0.73
261 : B00FQF0
S = −0.28
262 : B00FQJE
S = 0.09
263 : B00FQGH
S = −0.17
264 : B00EI55
S = −0.45
265 : B00FQI1
S = 0.08
266 : B00FQJF
S = −0.31
267 : B00FQG3
S = −0.49
268 : B00FQIC
S = 0.31
269 : B00EJL0
S = 0.23
270 : B00F6NR
S = −0.2
271 : B00FQIE
S = 0.05
272 : B00ER9E
S = −0.04
273 : B00EQZO
S = 0.01
274 : B00FQI7
S = 0.33
275 : B00FQF2
S = −0.07
276 : B00ER5K
S = 0.15
277 : B00FQI4
S = 0.33
278 : B00FQH1
S = −0.11
279 : B00EJLS
S = 0.04
280 : B00FQGJ
S = −0.2
281 : B00FQHL
S = 0.25
282 : B00EQXX
S = −0.68
283 : B00FQIN
S = −0.41
284 : B00EQUN
S = −0.43
285 : B00FQHF
S = 0.01
286 : B00ER5R
S = −0.51
287 : B00FQGB
S = −0.35
288 : B00ER96
S = 0.08
289 : B00ER7B
S = 0.06
290 : B00FQHI
S = 0.11
291 : B00FQI3
S = −0.19
292 : B00FQI9
S = −0.03
293 : B00FQGA
S = −0.13
294 : B00ER4A
S = −0.34
295 : B00EJKG
S = 0.09
296 : B00F6OH
S = 0.13
297 : B00ER5J
S = 0.17
298 : B00FQIB
S = 0.16
299 : B00ER0I
S = −0.1
300 : B00FQIY
S = −0.06
301 : B00ER6M
S = 0.13
302 : B00FQID
S = 0.02
303 : B00EJLC
S = 0.06
304 : B00FQIG
S = −0.02
305 : B00ER4J
S = −0.22
306 : B00FQGK
S = −0.39
307 : B00FQIH
S = 0.19
308 : B00EJKP
S = 0.08
309 : B00EQX3
S = 0.02
310 : B00FQIO
S = −0.08
311 : B00ER52
S = −0.28
312 : B00FQFO
S = 0.32
313 : B00EJKV
S = −0.54
314 : B00FQGS
S = −0.81
315 : B00EJL3
S = 0.17
316 : B00FQGM
S = −0.15
317 : B00FQGL
S = −0.45
318 : B00EQUP
S = −0.24
319 : B00EQZG
S = −0.57
320 : B00FQGN
S = −0.15
321 : B00EJJS
S = 0.06
322 : B00FQH5
S = −0.23
323 : B00FQIM
S = 0.23

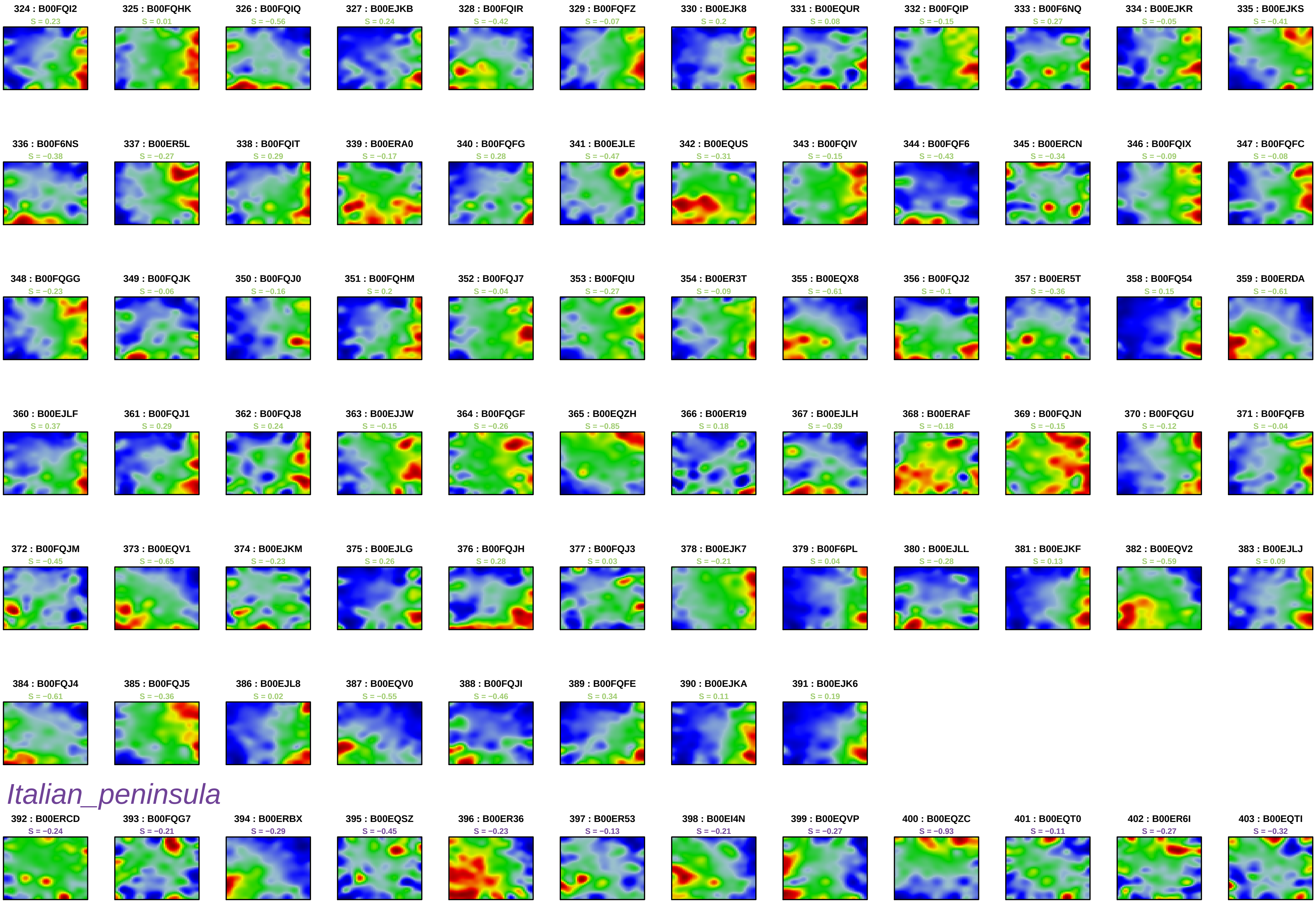

324 : B00FQI2
S = 0.23
325 : B00FQHK
S = 0.01
326 : B00FQIQ
S = −0.56
327 : B00EJKB
S = 0.24
328 : B00FQIR
S = −0.42
329 : B00FQFZ
S = −0.07
330 : B00EJK8
S = 0.2
331 : B00EQUR
S = 0.08
332 : B00FQIP
S = −0.15
333 : B00F6NQ
S = 0.27
334 : B00EJKR
S = −0.05
335 : B00EJKS
S = −0.41
336 : B00F6NS
S = −0.38
337 : B00ER5L
S = −0.27
338 : B00FQIT
S = 0.29
339 : B00ERA0
S = −0.17
340 : B00FQFG
S = 0.28
341 : B00EJLE
S = −0.47
342 : B00EQUS
S = −0.31
343 : B00FQIV
S = −0.15
344 : B00FQF6
S = −0.43
345 : B00ERCN
S = −0.34
346 : B00FQIX
S = −0.09
347 : B00FQFC
S = −0.08
348 : B00FQGG
S = −0.23
349 : B00FQJK
S = −0.06
350 : B00FQJ0
S = −0.16
351 : B00FQHM
S = 0.2
352 : B00FQJ7
S = −0.04
353 : B00FQIU
S = −0.27
354 : B00ER3T
S = −0.09
355 : B00EQX8
S = −0.61
356 : B00FQJ2
S = −0.1
357 : B00ER5T
S = −0.36
358 : B00FQ54
S = 0.15
359 : B00ERDA
S = −0.61
360 : B00EJLF
S = 0.37
361 : B00FQJ1
S = 0.29
362 : B00FQJ8
S = 0.24
363 : B00EJJW
S = −0.15
364 : B00FQGF
S = −0.26
365 : B00EQZH
S = −0.85
366 : B00ER19
S = 0.18
367 : B00EJLH
S = −0.39
368 : B00ERAF
S = −0.18
369 : B00FQJN
S = −0.15
370 : B00FQGU
S = −0.12
371 : B00FQFB
S = −0.04
372 : B00FQJM
S = −0.45
373 : B00EQV1
S = −0.65
374 : B00EJKM
S = −0.23
375 : B00EJLG
S = 0.26
376 : B00FQJH
S = 0.28
377 : B00FQJ3
S = 0.03
378 : B00EJK7
S = −0.21
379 : B00F6PL
S = 0.04
380 : B00EJLL
S = −0.28
381 : B00EJKF
S = 0.13
382 : B00EQV2
S = −0.59
383 : B00EJLJ
S = 0.09
384 : B00FQJ4
S = −0.61
385 : B00FQJ5
S = −0.36
386 : B00EJL8
S = 0.02
387 : B00EQV0
S = −0.55
388 : B00FQJI
S = −0.46
389 : B00FQFE
S = 0.34
390 : B00EJKA
S = 0.11
391 : B00EJK6
S = 0.19
Italian_peninsula
392 : B00ERCD
S = −0.24
393 : B00FQG7
S = −0.21
394 : B00ERBX
S = −0.29
395 : B00EQSZ
S = −0.45
396 : B00ER36
S = −0.23
397 : B00ER53
S = −0.13
398 : B00EI4N
S = −0.21
399 : B00EQVP
S = −0.27
400 : B00EQZC
S = −0.93
401 : B00EQT0
S = −0.11
402 : B00ER6I
S = −0.27
403 : B00EQTI
S = −0.32

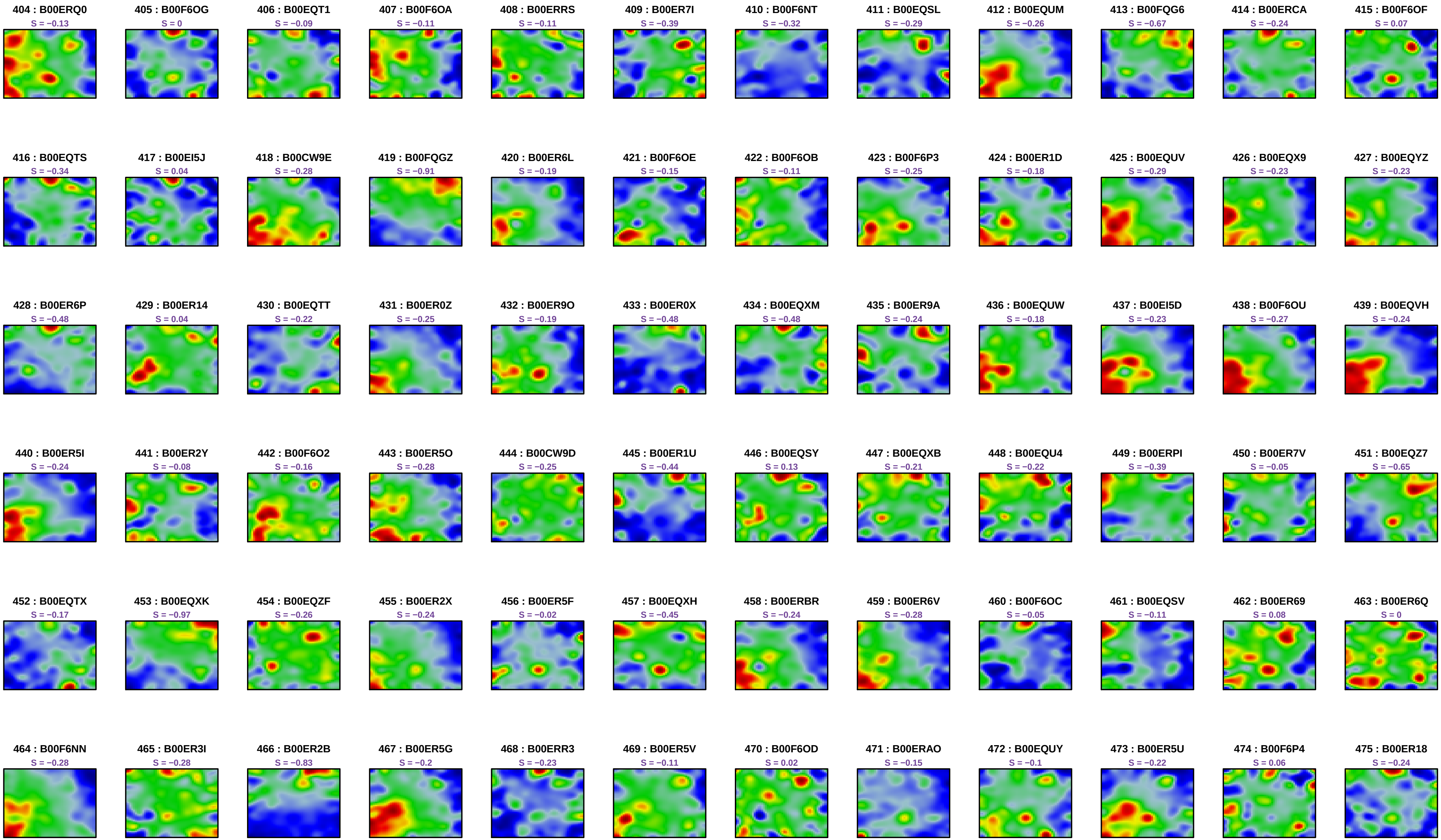

Middle_&_Far_East

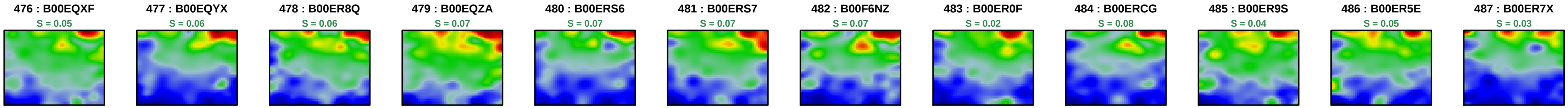

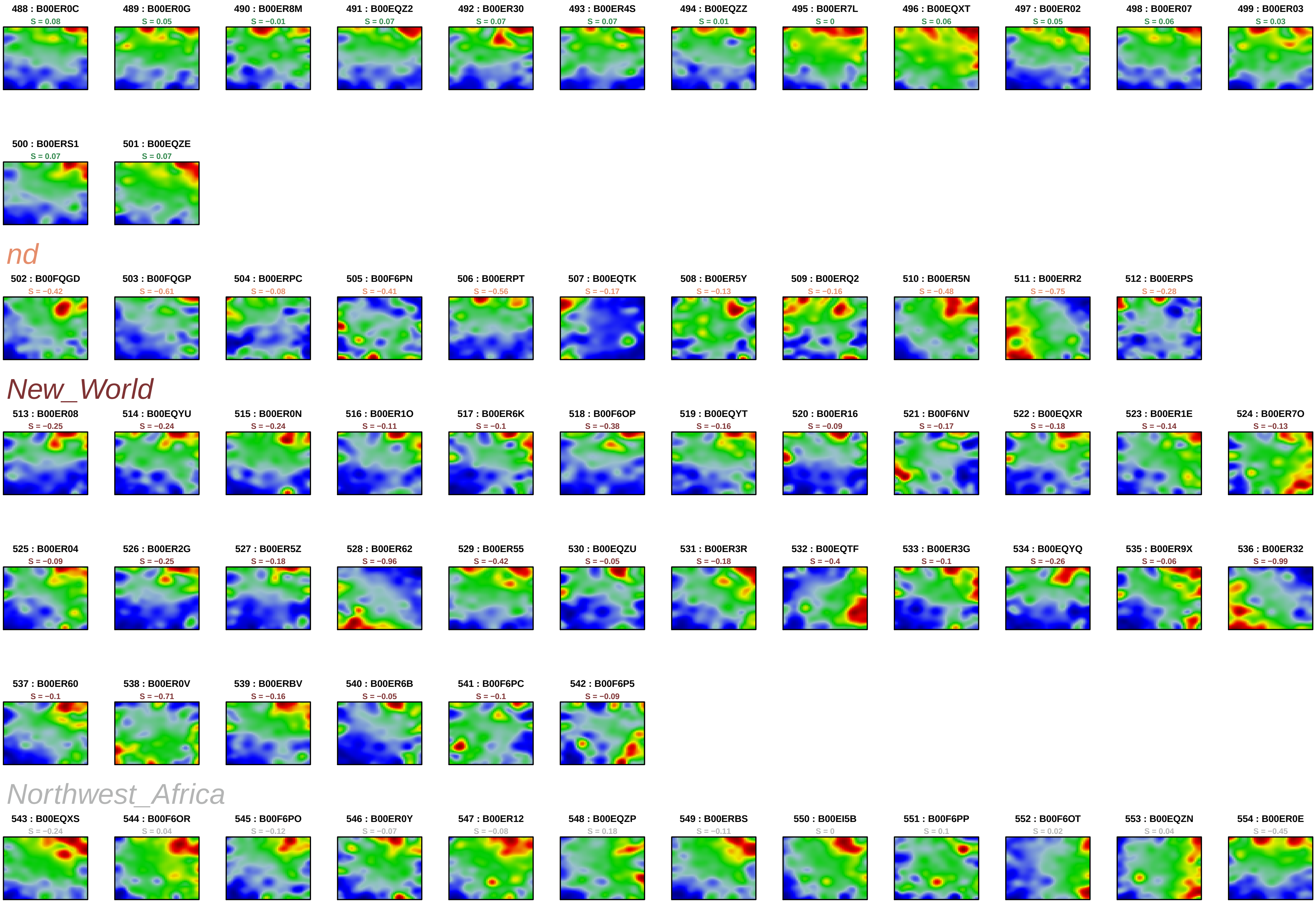

488 : B00ER0C
S = 0.08
489 : B00ER0G
S = 0.05
490 : B00ER8M
S = −0.01
491 : B00EQZ2
S = 0.07
492 : B00ER30
S = 0.07
493 : B00ER4S
S = 0.07
494 : B00EQZZ
S = 0.01
495 : B00ER7L
S = 0
496 : B00EQXT
S = 0.06
497 : B00ER02
S = 0.05
498 : B00ER07
S = 0.06
499 : B00ER03
S = 0.03
500 : B00ERS1
S = 0.07
501 : B00EQZE
S = 0.07
nd
502 : B00FQGD
S = −0.42
503 : B00FQGP
S = −0.61
504 : B00ERPC
S = −0.08
505 : B00F6PN
S = −0.41
506 : B00ERPT
S = −0.56
507 : B00EQTK
S = −0.17
508 : B00ER5Y
S = −0.13
509 : B00ERQ2
S = −0.16
510 : B00ER5N
S = −0.48
511 : B00ERR2
S = −0.75
512 : B00ERPS
S = −0.28
New_World
513 : B00ER08
S = −0.25
514 : B00EQYU
S = −0.24
515 : B00ER0N
S = −0.24
516 : B00ER1O
S = −0.11
517 : B00ER6K
S = −0.1
518 : B00F6OP
S = −0.38
519 : B00EQYT
S = −0.16
520 : B00ER16
S = −0.09
521 : B00F6NV
S = −0.17
522 : B00EQXR
S = −0.18
523 : B00ER1E
S = −0.14
524 : B00ER7O
S = −0.13
525 : B00ER04
S = −0.09
526 : B00ER2G
S = −0.25
527 : B00ER5Z
S = −0.18
528 : B00ER62
S = −0.96
529 : B00ER55
S = −0.42
530 : B00EQZU
S = −0.05
531 : B00ER3R
S = −0.18
532 : B00EQTF
S = −0.4
533 : B00ER3G
S = −0.1
534 : B00EQYQ
S = −0.26
535 : B00ER9X
S = −0.06
536 : B00ER32
S = −0.99
537 : B00ER60
S = −0.1
538 : B00ER0V
S = −0.71
539 : B00ERBV
S = −0.16
540 : B00ER6B
S = −0.05
541 : B00F6PC
S = −0.1
542 : B00F6P5
S = −0.09
Northwest_Africa
543 : B00EQXS
S = −0.24
544 : B00F6OR
S = 0.04
545 : B00F6PO
S = −0.12
546 : B00ER0Y
S = −0.07
547 : B00ER12
S = −0.08
548 : B00EQZP
S = 0.18
549 : B00ERBS
S = −0.11
550 : B00EI5B
S = 0
551 : B00F6PP
S = 0.1
552 : B00F6OT
S = 0.02
553 : B00EQZN
S = 0.04
554 : B00ER0E
S = −0.45

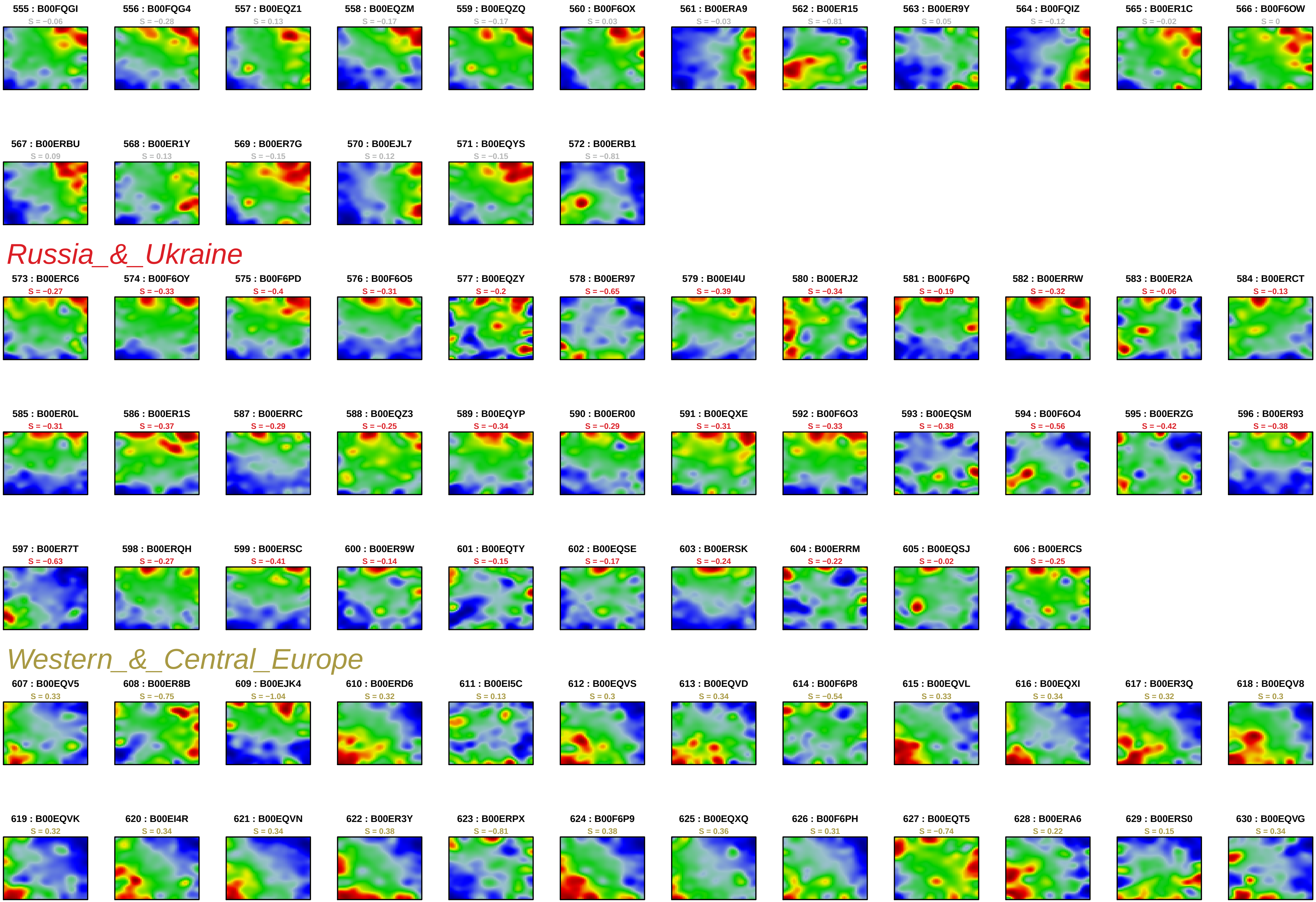

555 : B00FQGI
S = −0.06
556 : B00FQG4
S = −0.28
557 : B00EQZ1
S = 0.13
558 : B00EQZM
S = −0.17
559 : B00EQZQ
S = −0.17
560 : B00F6OX
S = 0.03
561 : B00ERA9
S = −0.03
562 : B00ER15
S = −0.81
563 : B00ER9Y
S = 0.05
564 : B00FQIZ
S = −0.12
565 : B00ER1C
S = −0.02
566 : B00F6OW
S = 0
567 : B00ERBU
S = 0.09
568 : B00ER1Y
S = 0.13
569 : B00ER7G
S = −0.15
570 : B00EJL7
S = 0.12
571 : B00EQYS
S = −0.15
572 : B00ERB1
S = −0.81
Russia_&_Ukraine
573 : B00ERC6
S = −0.27
574 : B00F6OY
S = −0.33
575 : B00F6PD
S = −0.4
576 : B00F6O5
S = −0.31
577 : B00EQZY
S = −0.2
578 : B00ER97
S = −0.65
579 : B00EI4U
S = −0.39
580 : B00ERJ2
S = −0.34
581 : B00F6PQ
S = −0.19
582 : B00ERRW
S = −0.32
583 : B00ER2A
S = −0.06
584 : B00ERCT
S = −0.13
585 : B00ER0L
S = −0.31
586 : B00ER1S
S = −0.37
587 : B00ERRC
S = −0.29
588 : B00EQZ3
S = −0.25
589 : B00EQYP
S = −0.34
590 : B00ER00
S = −0.29
591 : B00EQXE
S = −0.31
592 : B00F6O3
S = −0.33
593 : B00EQSM
S = −0.38
594 : B00F6O4
S = −0.56
595 : B00ERZG
S = −0.42
596 : B00ER93
S = −0.38
597 : B00ER7T
S = −0.63
598 : B00ERQH
S = −0.27
599 : B00ERSC
S = −0.41
600 : B00ER9W
S = −0.14
601 : B00EQTY
S = −0.15
602 : B00EQSE
S = −0.17
603 : B00ERSK
S = −0.24
604 : B00ERRM
S = −0.22
605 : B00EQSJ
S = −0.02
606 : B00ERCS
S = −0.25
Western_&_Central_Europe
607 : B00EQV5
S = 0.33
608 : B00ER8B
S = −0.75
609 : B00EJK4
S = −1.04
610 : B00ERD6
S = 0.32
611 : B00EI5C
S = 0.13
612 : B00EQVS
S = 0.3
613 : B00EQVD
S = 0.34
614 : B00F6P8
S = −0.54
615 : B00EQVL
S = 0.33
616 : B00EQXI
S = 0.34
617 : B00ER3Q
S = 0.32
618 : B00EQV8
S = 0.3
619 : B00EQVK
S = 0.32
620 : B00EI4R
S = 0.34
621 : B00EQVN
S = 0.34
622 : B00ER3Y
S = 0.38
623 : B00ERPX
S = −0.81
624 : B00F6P9
S = 0.38
625 : B00EQXQ
S = 0.36
626 : B00F6PH
S = 0.31
627 : B00EQT5
S = −0.74
628 : B00ERA6
S = 0.22
629 : B00ERS0
S = 0.15
630 : B00EQVG
S = 0.34

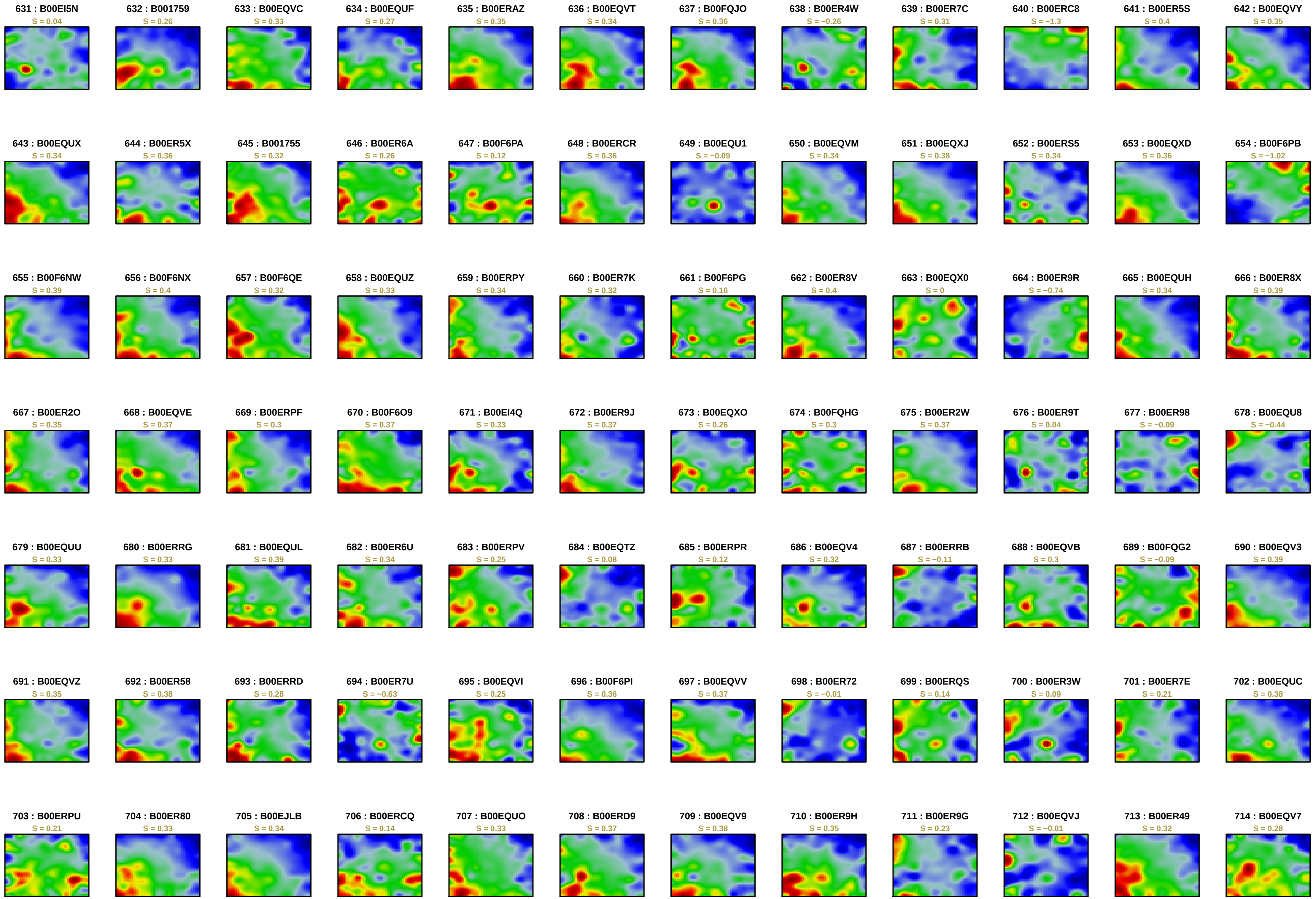
631 : B00EI5N
S = 0.04
632 : B001759
S = 0.26
633 : B00EQVC
S = 0.33
634 : B00EQUF
S = 0.27
635 : B00ERAZ
S = 0.35
636 : B00EQVT
S = 0.34
637 : B00FQJO
S = 0.36
638 : B00ER4W
S = −0.26
639 : B00ER7C
S = 0.31
640 : B00ERC8
S = −1.3
641 : B00ER5S
S = 0.4
642 : B00EQVY
S = 0.35
643 : B00EQUX
S = 0.34
644 : B00ER5X
S = 0.36
645 : B001755
S = 0.32
646 : B00ER6A
S = 0.26
647 : B00F6PA
S = 0.12
648 : B00ERCR
S = 0.36
649 : B00EQU1
S = −0.09
650 : B00EQVM
S = 0.34
651 : B00EQXJ
S = 0.38
652 : B00ERS5
S = 0.34
653 : B00EQXD
S = 0.36
654 : B00F6PB
S = −1.02
655 : B00F6NW
S = 0.39
656 : B00F6NX
S = 0.4
657 : B00F6QE
S = 0.32
658 : B00EQUZ
S = 0.33
659 : B00ERPY
S = 0.34
660 : B00ER7K
S = 0.32
661 : B00F6PG
S = 0.16
662 : B00ER8V
S = 0.4
663 : B00EQX0
S = 0
664 : B00ER9R
S = −0.74
665 : B00EQUH
S = 0.34
666 : B00ER8X
S = 0.39
667 : B00ER2O
S = 0.35
668 : B00EQVE
S = 0.37
669 : B00ERPF
S = 0.3
670 : B00F6O9
S = 0.37
671 : B00EI4Q
S = 0.33
672 : B00ER9J
S = 0.37
673 : B00EQXO
S = 0.26
674 : B00FQHG
S = 0.3
675 : B00ER2W
S = 0.37
676 : B00ER9T
S = 0.04
677 : B00ER98
S = −0.09
678 : B00EQU8
S = −0.44
679 : B00EQUU
S = 0.33
680 : B00ERRG
S = 0.33
681 : B00EQUL
S = 0.39
682 : B00ER6U
S = 0.34
683 : B00ERPV
S = 0.25
684 : B00EQTZ
S = 0.08
685 : B00ERPR
S = 0.12
686 : B00EQV4
S = 0.32
687 : B00ERRB
S = −0.11
688 : B00EQVB
S = 0.3
689 : B00FQG2
S = −0.09
690 : B00EQV3
S = 0.39
691 : B00EQVZ
S = 0.35
692 : B00ER58
S = 0.38
693 : B00ERRD
S = 0.28
694 : B00ER7U
S = −0.63
695 : B00EQVI
S = 0.25
696 : B00F6PI
S = 0.36
697 : B00EQVV
S = 0.37
698 : B00ER72
S = −0.01
699 : B00ERQS
S = 0.14
700 : B00ER3W
S = 0.09
701 : B00ER7E
S = 0.21
702 : B00EQUC
S = 0.38
703 : B00ERPU
S = 0.21
704 : B00ER80
S = 0.33
705 : B00EJLB
S = 0.34
706 : B00ERCQ
S = 0.14
707 : B00EQUO
S = 0.33
708 : B00ERD9
S = 0.37
709 : B00EQV9
S = 0.38
710 : B00ER9H
S = 0.35
711 : B00ER9G
S = 0.23
712 : B00EQVJ
S = −0.01
713 : B00ER49
S = 0.32
714 : B00EQV7
S = 0.28

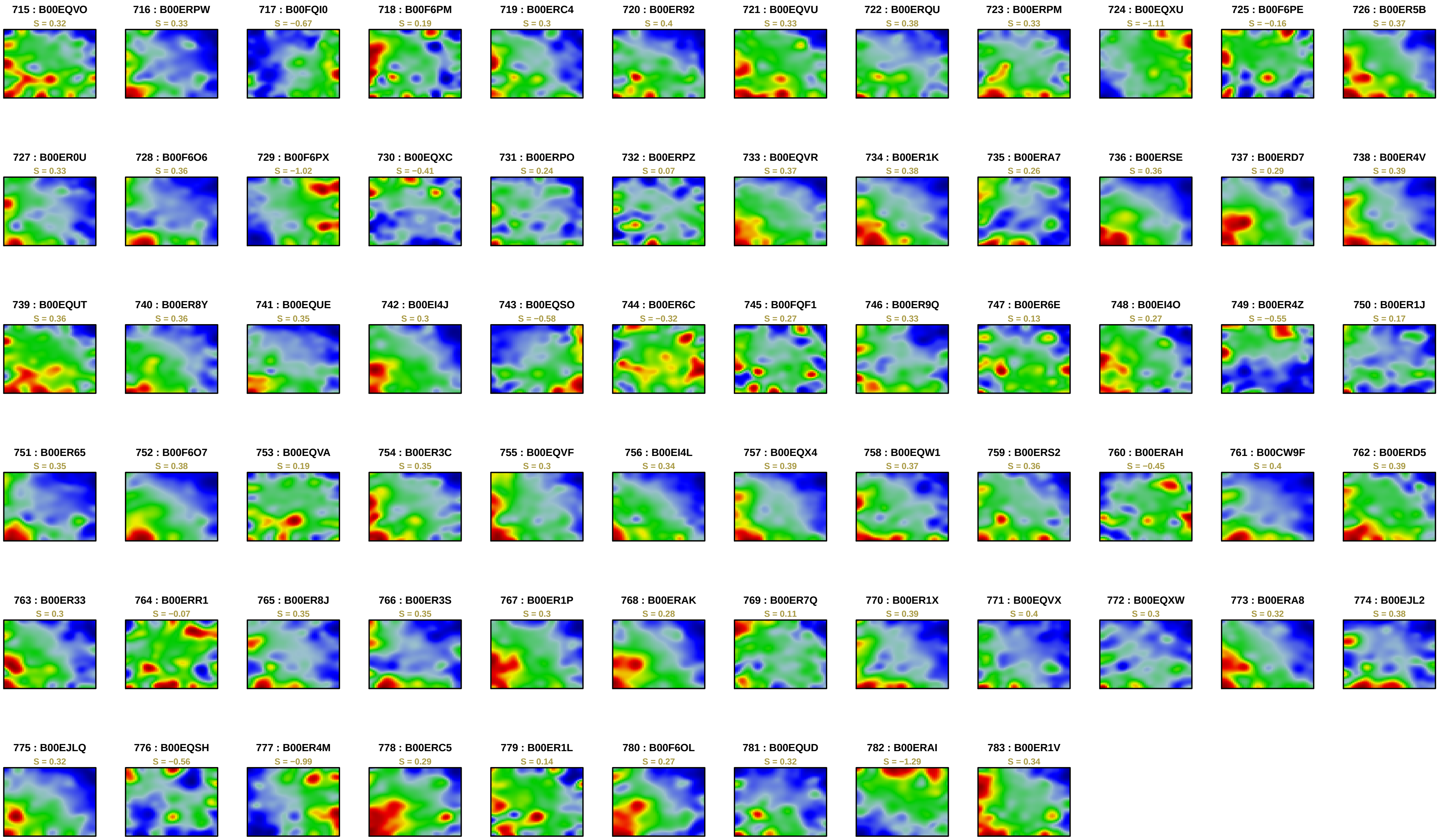
715 : B00EQVO
S = 0.32
716 : B00ERPW
S = 0.33
717 : B00FQI0
S = −0.67
718 : B00F6PM
S = 0.19
719 : B00ERC4
S = 0.3
720 : B00ER92
S = 0.4
721 : B00EQVU
S = 0.33
722 : B00ERQU
S = 0.38
723 : B00ERPM
S = 0.33
724 : B00EQXU
S = −1.11
725 : B00F6PE
S = −0.16
726 : B00ER5B
S = 0.37
727 : B00ER0U
S = 0.33
728 : B00F6O6
S = 0.36
729 : B00F6PX
S = −1.02
730 : B00EQXC
S = −0.41
731 : B00ERPO
S = 0.24
732 : B00ERPZ
S = 0.07
733 : B00EQVR
S = 0.37
734 : B00ER1K
S = 0.38
735 : B00ERA7
S = 0.26
736 : B00ERSE
S = 0.36
737 : B00ERD7
S = 0.29
738 : B00ER4V
S = 0.39
739 : B00EQUT
S = 0.36
740 : B00ER8Y
S = 0.36
741 : B00EQUE
S = 0.35
742 : B00EI4J
S = 0.3
743 : B00EQSO
S = −0.58
744 : B00ER6C
S = −0.32
745 : B00FQF1
S = 0.27
746 : B00ER9Q
S = 0.33
747 : B00ER6E
S = 0.13
748 : B00EI4O
S = 0.27
749 : B00ER4Z
S = −0.55
750 : B00ER1J
S = 0.17
751 : B00ER65
S = 0.35
752 : B00F6O7
S = 0.38
753 : B00EQVA
S = 0.19
754 : B00ER3C
S = 0.35
755 : B00EQVF
S = 0.3
756 : B00EI4L
S = 0.34
757 : B00EQX4
S = 0.39
758 : B00EQW1
S = 0.37
759 : B00ERS2
S = 0.36
760 : B00ERAH
S = −0.45
761 : B00CW9F
S = 0.4
762 : B00ERD5
S = 0.39
763 : B00ER33
S = 0.3
764 : B00ERR1
S = −0.07
765 : B00ER8J
S = 0.35
766 : B00ER3S
S = 0.35
767 : B00ER1P
S = 0.3
768 : B00ERAK
S = 0.28
769 : B00ER7Q
S = 0.11
770 : B00ER1X
S = 0.39
771 : B00EQVX
S = 0.4
772 : B00EQXW
S = 0.3
773 : B00ERA8
S = 0.32
774 : B00EJL2
S = 0.38
775 : B00EJLQ
S = 0.32
776 : B00EQSH
S = −0.56
777 : B00ER4M
S = −0.99
778 : B00ERC5
S = 0.29
779 : B00ER1L
S = 0.14
780 : B00F6OL
S = 0.27
781 : B00EQUD
S = 0.32
782 : B00ERAI
S = −1.29
783 : B00ER1V
S = 0.34
