# Supplementary material for: SOMmelier—Intuitive Visualization of the Topology of Grapevine Genome Landscapes Using Artificial Neural Networks
Source: Genes (Basel). 2020 Jul 17;11(7):817. doi: 10.3390/genes11070817 (PMC7397337; doi:10.3390/genes11070817)
Supplement: Supplementary file 1 [file genes-11-00817-s001.zip › Additional File 4.docx]

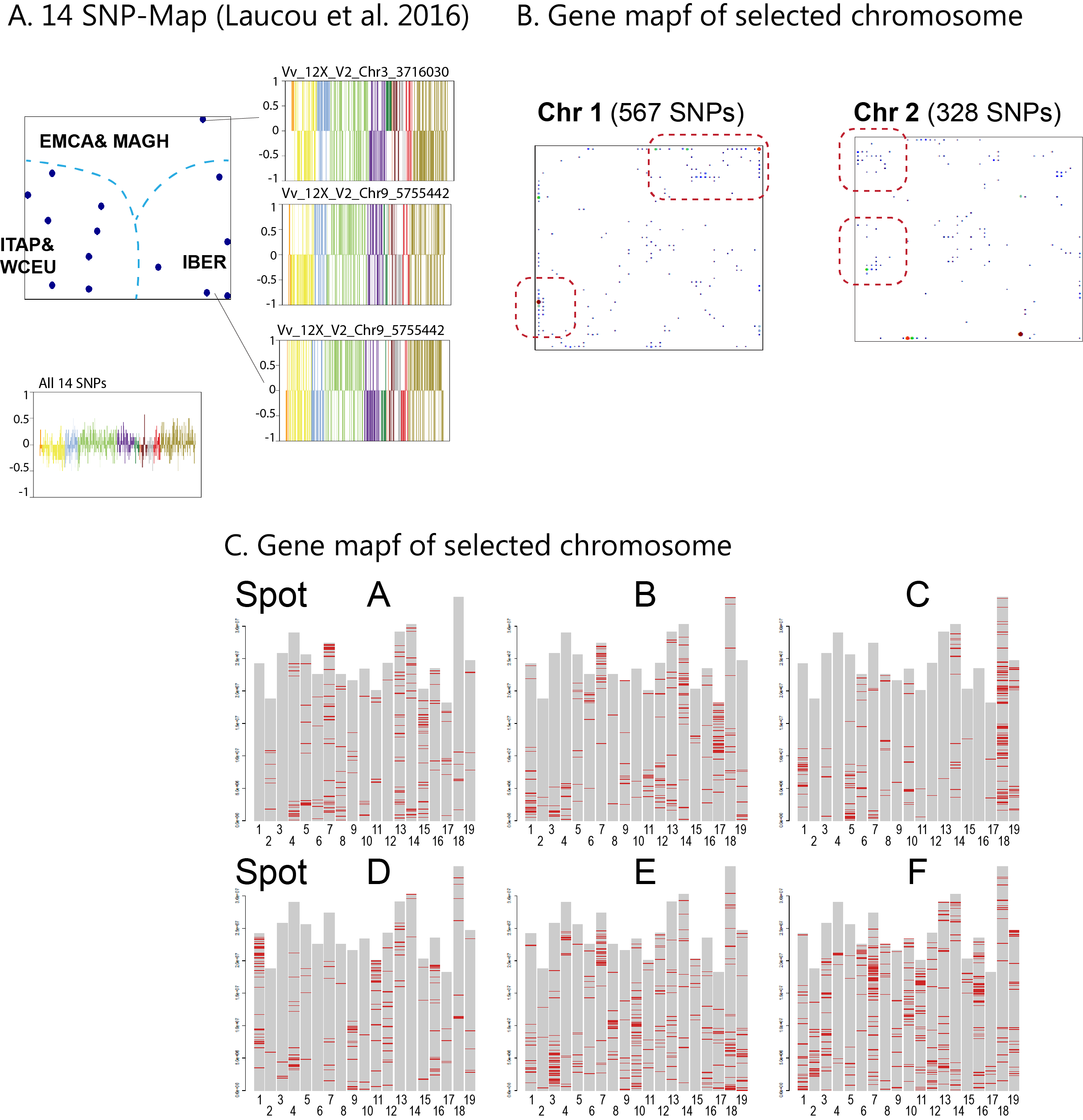


**Figure S1:** SNP maps, eMAF profiles and associations with selected phenotypes. (a) 14 SNPs (assembly version 12X.V0) selected in [11] to identify the 783 vine cultivars accumulate in WCEU/ITAP and IBER regions of the map. Their mean SNP profile shows large eMAF-values for cultivars from these regions on the average. Profiles of selected individual SNPs reveal a nearly bi-modal distribution of eMAF-values. (b) SNPs from different chromosomes distribute over several spot regions (red frames). (c) Distribution of SNPs from selected spots (red marks) among the 19 chromosomes (grey vertical bars).


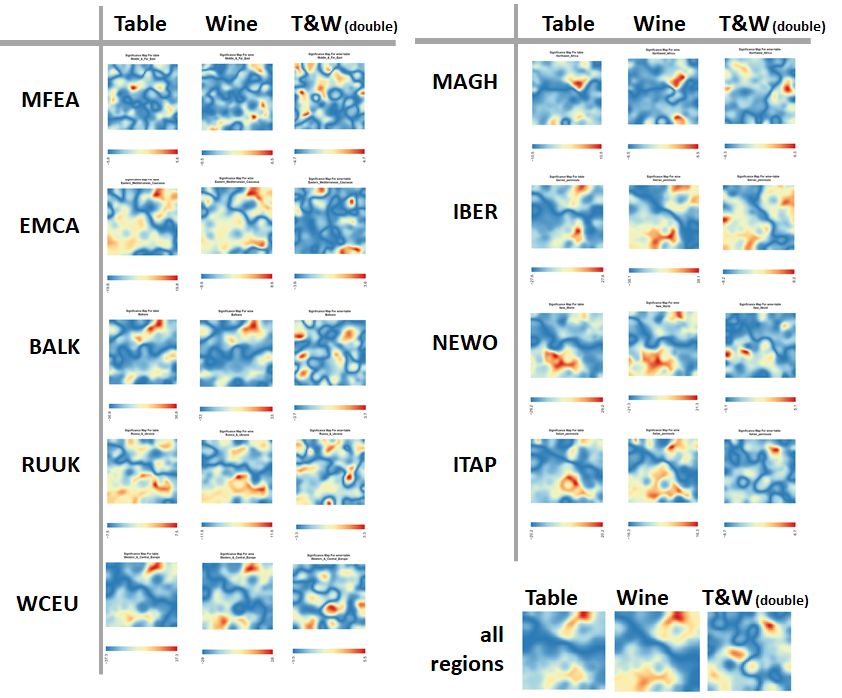


**Figure S2:** ANOVA-portraits stratified according to geographical regions and utilization of grapes. The log P-value is visualized on the SOM map. The most significant areas for all phenotypes overlap with characteristic spots for MAGH cultivars.

**References**

see main paper
